# Transcriptomic and biochemical analysis of pummelo x finger lime hybrids in response to Huanglongbing (HLB)

**DOI:** 10.1186/s12870-025-06211-8

**Published:** 2025-02-20

**Authors:** Lamiaa M. Mahmoud, Jaideep Kaur Deol, Jude W. Grosser, Nabil Killiny, Manjul Dutt

**Affiliations:** 1 Department of Horticultural Sciences, Citrus Research and Education Center, Institute of Food and Agricultural Sciences, University of Florida, Lake Alfred, FL 33850 USA; 2 Department of Plant Pathology, Citrus Research and Education Center, IFAS, University of Florida, Lake Alfred, FL 33850 USA; 3 Plant Breeding Graduate Program, University of Florida, Gainesville, FL 32611 USA

**Keywords:** Citrus greening, Huanglongbing (HLB), Finger lime hybrids, Conventional breeding, *Candidatus* liberibacter asiaticus (*Ca*Las), Transcriptomic analysis

## Abstract

**Background:**

Huanglongbing (HLB) is a devastating bacterial disease caused by the bacterium *Candidatus* Liberibacter asiaticus (*Ca*Las) that affects the citrus industry worldwide. This study investigated the response of two pummelo x finger lime hybrid siblings to natural infection with *Ca*Las. The hybrids were identified primarily using leaf morphology and molecular marker assessments and were selected for further studies on the basis of the *Ca*Las titers in leaf petioles.

**Results:**

HLB-infected budwood from the selected hybrids (PFL 2–61 and PFL 1–11), as well as the two parental plants, were propagated by grafting onto Swingle citrumelo rootstocks for further evaluation. Plant samples were collected two years after grafting for analysis. Leaves of PFL2-61 exhibited decreased *Ca*Las titers compared with those of PFL 1–11. Additionally, we recorded increased chlorophyll content, total phenolic content (TPC), total flavonoid content (TFC), and antioxidant activity in PFL 2–61 compared to PFL 1–11 and the parents. We subsequently conducted a detailed investigation of these two hybrid siblings using transcriptome analysis. Among the 20,675 differentially expressed genes (DEGs) identified, 1,416 were downregulated in PFL 2–61 compared with PFL 1–11, whereas 326 were upregulated. Transcriptome analysis revealed that many of the DEGs were associated with the cell wall structure, redox homeostasis, and biotic stress responses. Moreover, key genes related to the biosynthesis of secondary metabolites and phytohormones, including *PAL1*, jasmonate-related genes, and *WRKY* transcription factors, were upregulated in the tolerant hybrid (PFL 2–61). In contrast, three transcripts associated with the Sieve Element Occlusion N-Terminus (*SEO_N*) domain were downregulated in the tolerant hybrid (PFL 2–61).

**Conclusions:**

Our findings provide valuable insights into the molecular mechanisms of tolerance and susceptibility to HLB in finger lime derived hybrids, highlighting the potential of this citrus species towards developing disease-tolerant varieties.

**Graphical Abstract:**

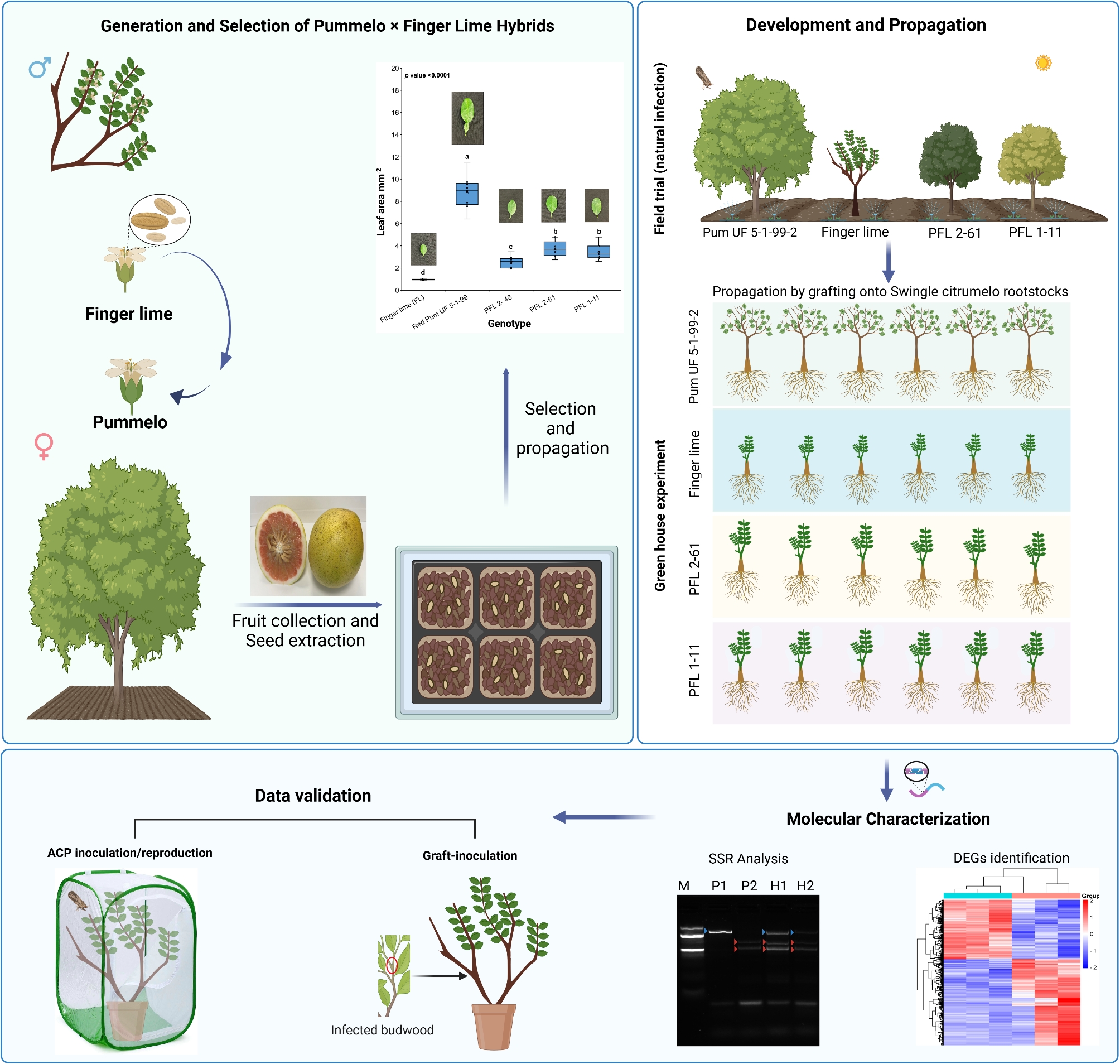

**Supplementary Information:**

The online version contains supplementary material available at 10.1186/s12870-025-06211-8.

## Background

Citrus is a major global fruit crop that is consumed for its high abundance of carbohydrates, vitamins (especially vitamin C), minerals, carotenoids, flavonoids, and dietary fibers [1]. Currently, the major challenge facing the citrus industry is citrus greening disease, commonly known as Huanglongbing (HLB). HLB causes a substantial threat to global citrus production, presenting significant economic and agricultural challenges [2]. HLB is caused by a gram-negative bacterium called *Candidatus* Liberibacter asiaticus (*Ca*Las), transmitted by the Asian citrus psyllid *Diaphorina citri*. Infection leads to severe disruption of phloem function and impairment of nutrient transport within host plants [3]. *Ca*Las manipulates its hosts by modulating host physiology, suppressing plant defense mechanisms, and creating environmental conditions conducive to colonization and proliferation [4, 5]. Since its initial detection in 2005, HLB has rapidly spread throughout Florida, affecting every citrus-growing county [6]. Furthermore, HLB has emerged in Texas and California, threatening the crucial Central Valley [7, 8].

Mitigating HLB in citrus orchards requires a multifaceted strategy due to the complex nature of the disease. Vector control, nutritional management, thermotherapy, and antimicrobial and antibiotic applications have been utilized to control *Ca*Las infection. However, these strategies have shown limited success in field applications, highlighting the challenge of effective long-term HLB management. Thus, breeding for HLB-tolerant varieties may be the most effective and sustainable solution [9, 10].

Studies have been conducted to identify specific genes and proteins activated or expressed in leaf tissues in response to HLB [11, 12]. In general, plant pathogens induce the production of a diverse array of defense compounds, including phenolic compounds, terpenoids, alkaloids, nonprotein amino acids, benzoxazinoids, and cyanogenic glucosides, which are produced as a part of an intercellular defensive response [13]. These compounds are often accompanied by the synthesis of pathogenesis-related proteins, contributing to a systemic acquired immune response [14].

Although most commercially grown citrus cultivars are highly susceptible to HLB, several studies have reported HLB tolerance in certain citrus species. The most known species for their tolerance are citron species (e.g., lemons) and their hybrids, *C. latipes* [15, 16], and *Poncirus trifoliate* and their hybrids [17, 18]. Among these species, *Citrus glauca*, *Citrus australasica*, and the hybrid of *Citrus australis* and *Citrus virgata* (Sydney hybrid) have exhibited resistance or high tolerance to HLB [10, 12, 19, 20]. In our recent study, we identified several HLB tolerance traits in Australian lime species that present opportunities for incorporating these genes to increase HLB tolerance in traditional citrus cultivars [12]. The observed tolerance of finger limes may be related to vector preference, as finger lime trees can be infected under controlled conditions following budding with *Ca*Las-infected budwood [12, 21]. Moreover, *Diaphorina citri* shows a feeding preference for certain citrus cultivars, with ‘Valencia’ sweet orange considered a preferred host, while finger limes exhibited less preference [22].

Numerous red-fleshed pummelo species are available in the germplasm collection at the Citrus Research and Education Center (CREC), Lake Alfred, FL for breeding [23]. The use of red-fleshed tetraploid pummelos as the female parent in crosses has the potential to create hybrids that are more attractive and appealing to consumers [24]. HLB-tolerant pummelo interstocks could enhance defense mechanisms and improve citrus tree growth and tolerance to HLB infection in ‘Valencia’ scions [25]. In our plant breeding program at the University of Florida, we utilized finger limes in two main ways: (*i*) to create new citrus-like cultivars suitable for use as rootstocks, interstocks, or scions and (*ii*) to identify and map the genes responsible for HLB tolerance to facilitate direct modification of established elite citrus cultivars via modern biotechnology tools, particularly those that are challenging to modify through traditional breeding due to their high heterozygosity.

In the present study, we crossed a select red-fleshed pummelo with the finger lime, and from a large field population compared two selected F1 pummelo × finger lime hybrids with varying responses to HLB. By comparing the transcriptomic responses of these hybrids, we aim to investigate genetic factors and molecular pathways that contribute to HLB tolerance. The hybrids that were subjected to field infection were also clonally propagated for evaluation by grafting in the greenhouse, followed by analysis of differentially expressed genes (DEGs). Additionally, biochemical assays were conducted to assess plant traits such as chlorophyll content, total phenolic compounds, and flavonoid content. A separate group of clonally propagated hybrids (produced from cuttings) was assessed after *Ca*Las infection using a no-choice ACP (*Diaphorina citri*) feeding assay to validate the DEGs.

## Methods

### Plant materials and growth conditions

Pummelo x finger lime hybrids were developed by crossing a select red pulp pummelo (Red Pum UF 5-1-99-2, an open pollinated seedling selection of the Hirado Buntan pummelo; HBP) with the finger lime (PI 312872 accession from the USDA National Plant Germplasm System (NPGS)). The selection process for the first generation (F1) involved visual analysis of leaf size and determining the presence of anthocyanin in the new leaves. Genomic DNA was extracted from fresh leaves via the GeneJET Plant Genomic DNA Purification Mini Kit, and 111 SSR primers were evaluated to identify polymorphic molecular markers associated with specific crosses. Polymorphic primers were subsequently tested on individual crosses and their hybrids to validate hybrid purity [26]. Reaction mixtures for PCR with a total volume of 10 µL were prepared using genomic DNA, primers, and Promega GoTaq™ G2 Green Master Mix, with amplification conditions optimized on an MJ RESEARCH Peltier Thermal Cycler. The amplified products were resolved on a 3.5% agarose gel [27] and visualized using an AXYGEN gel documentation system. A total of 24 polymorphic primers were developed for the Pum UF 5-1-99-2 × finger lime (PI 312872) cross, with five specific primers developed to confirm hybrid purity. Most SSR markers had amplicon sizes ranging from 100 to 250 bp (Tables S1 and Figure S1). The selected hybrids were planted for 8–10 years in a sandy-soil field in Lake Alfred, Florida, USA, where they were exposed to natural psyllid feeding. The trees were irrigated daily, and Florikan 12-4-8 360-day controlled-release fertilizer (FLORIKAN E.S.A. LLC, Sarasota, Florida) was applied annually in March. Twelve budwoods were collected from the field-grown hybrids and their parent trees. The collected budwoods were grafted onto Swingle citrumelo rootstocks. The plants were then maintained in a greenhouse with a relative humidity of 70 ± 10% and a temperature of 32 ± 2 °C, under a natural 16-hour photoperiod and 50% shading. Ten randomly selected, pest- and disease-free mature leaves were collected from two-year-old propagated trees. The leaves were placed on uniform A4 paper, scanned, and analyzed using ImageJ software to calculate the average leaf area.

### Monitoring *Ca*Las infection in pummelo and finger lime hybrids

To assess the *Ca*Las titer in greenhouse-grown trees, genomic DNA was isolated from the leaf petioles and midveins of fully expanded leaves in late fall of 2022. The trees were maintained in a greenhouse and evaluated for the presence of *Ca*Las 12 months after infection. DNA was extracted via a GeneJET Plant Genomic DNA Purification Kit from Thermo Fisher Scientific (Waltham, MA, USA). The DNA concentration was subsequently standardized to 25 ng/µL before quantitative PCR (qPCR) was performed. The detection of *Ca*Las genomic DNA was performed through qPCR, employing TaqMan™ Gene Expression Master Mix and CQUL primers targeting the *Ca*Las rplJ/rplL ribosomal protein-encoding gene, following the protocol outlined by Wang et al. [28]. To quantify the quantity of *Ca*Las, a standard curve was generated using plasmids containing the target sequence. Plasmids containing amplicons were diluted to a final concentration of 10 ng/µL. DNA samples were run alongside standard solutions previously prepared by performing serial dilutions for analysis. The dilutions ranged from 1 × 10^3 to 1 × 10^9 copies of the target amplicon. This analysis was carried out using a QuantStudio™ 3 System.

### Biochemical parameter measurements

The leaves were collected and frozen in liquid nitrogen, followed by fine grinding. Six biological replicates were sampled from each tree, and the ground leaves were stored at − 20 °C for biochemical assays. For chlorophyll analysis, a total of 100 mg fresh weight was homogenized in 1 mL of absolute methanol according to Lichtenthaler and Wellburn [29]. The foliar phenolic compound content (TPC) was estimated using the Folin–Ciocalteu method with modifications [30]. The TPC extract was centrifuged, and 100 µL of Folin reagent (1:10) was mixed with the leaf extract. After vortexing and 5 min of incubation at room temperature, the reaction was initiated by adding 300 mL of 20% sodium carbonate (Na_2_CO_3_) to the extract. The tubes were then incubated in the dark for 1 h, and the absorbance was measured at 765 nm. A standard curve was generated using gallic acid standard solutions (0–600 ppm).

The total flavonoid content in the leaf samples was estimated using a colorimetric assay with aluminum chloride [31]. The methanolic extract (50 µL) was diluted in 200 µL of distilled water, and the diluted samples were mixed with 60 µL of 5% sodium nitrite (NaNO_2_) solution and 60 µL of 10% aluminum chloride (AlCl_3_) solution. After a 5-minute incubation at room temperature, 400 µL of 1 M sodium hydroxide (NaOH) and 1.230 mL of distilled water were added before vortexing. The absorbance of the reaction mixture was measured at 510 nm. A standard curve was created using catechin standard solutions (0–200 ppm), and the content of total flavonoids was expressed as mg catechin g^− 1^ per 100 mg fresh weight (FW).

### RNA isolation, cDNA library construction, and RNA sequencing (RNA-seq)

RNA was extracted from two-year-old growing trees in the greenhouse via TRIzol^®^ following the manufacturer’s protocol. High-quality RNA samples from three biological replicates derived from independent trees were used for cDNA synthesis and RNA-seq for the selected hybrids (PFL 1–11 and PFL 2–61). The RNA quality and quantity were analyzed using a NanoDrop™ One/OneC Microvolume UV‒Vis Spectrophotometer (Thermo Scientific). RNA samples with an RNA integrity number > 6.5 were selected for RNA sequencing. Total RNA was treated with oligo(dT) beads to enrich mRNAs with a poly(A) tail. Furthermore, mRNA molecules were fragmented into small pieces, and the fragmented RNA was synthesized into first-strand cDNA via random primers. The synthesized cDNA underwent end repair and 3’ adenylation. These 3’ adenylated cDNA fragments were ligated with adaptors and digested using a U-labeled second-strand template with uracil-DNA-glycosylase to perform PCR amplification.

Raw sequence reads in FASTQ format was subjected to the removal of adaptor sequences and filtering. A series of data processing steps were performed to remove contamination and obtain valid data via the SOAPnuke software developed by BGI [32]. A filter read length of 150 bp or more and an N content of 0.1% or more were selected, and less than the set parameters were discarded from the data. Low-quality data were eliminated if the bases had a read count quality value of < 20. The cleaned reads were mapped to the *Citrus clementina* genome, and the annotations were obtained from Phytozome (https://phytozome-next.jgi.doe.gov/) and aligned via Bowtie2 software. A metadata file for comparison was also generated for differentially expressed gene (DEG) analysis via DESeq2 v3.10, an R Bioconductor package [33]. Only significant DEGs whose *p* value was < 0.05 were identified for further analysis. The statistically significant Gene Ontology (GO) terms were input into REVIGO software to remove the redundant terms [34]. The functional pathway analysis was performed via the pathway function of MapMan (MapMan version 3.5.0) [35]. The functional categories were viewed via PageMan and analyzed for statistical significance via a nonparametric test [36].

### Quantitative PCR and DEG validation

The qPCR mixture consisted of 1 µL of DNA (25 ng/µL), SYBR^®^ Green PowerUp™ PCR Master Mix (Applied Biosystems, Foster City, CA), and gene-specific primers (Integrated DNA Technologies, Inc., Coralville, IA, USA) in a final volume of 20 µL, as per the manufacturer’s instructions. The analysis was performed in a StepOnePlus™ Real-Time PCR System (Thermo Fisher Scientific, Massachusetts, USA). The relative mRNA levels were compared to those of the endogenous ACTIN gene via the same 2^−ΔΔCT^ method [37]. To validate the DEGs, four upregulated and four downregulated genes were selected from the DEG data for qRT-PCR verification to test the reliability of the transcriptome data from the same samples that were sequenced. For further validation following *Ca*Las infection, the clonally propagated hybrids were infected with *Ca*Las bacteria through a no-choice ACP (*Diaphorina citri*) feeding assay or by grafting infected materials. Furthermore, some DEGs exhibiting significant differences were validated in parent and hybrid samples collected from one-year-old infected trees by forced infection via ACP feeding or grafting to infected materials. The relative gene expression of these trees was compared with that of finger lime trees. A list of the primers used in this study is presented in Table S2.

### Statistical analysis

All the statistical analyses were performed via JMP Pro v16 software. The hybrids and their parents were compared via analysis of variance (ANOVA) followed by Tukey–Kramer honestly significant difference (HSD) post hoc tests. A two-tailed *t-*test was used for pairwise statistical comparisons between the two hybrids, PFL 2–61 and PFL 1–11. Statistical significance was established at *p* < 0.05. The Pearson correlation coefficient (r) was calculated to validate the modulation in gene expression for the RNA-seq data and quantitative PCR. Two-way hierarchical cluster analysis (HCA) associated with heatmaps was performed using the values of the transcript levels of various genes involved in different pathways (three repeats for each hybrid). Distance and linkage of HCA were conducted via Ward’s minimum variance method [38], with 95% confidence between groups from the discriminant function analysis to construct the similarity dendrograms.

## Results

**Fig. 1:**
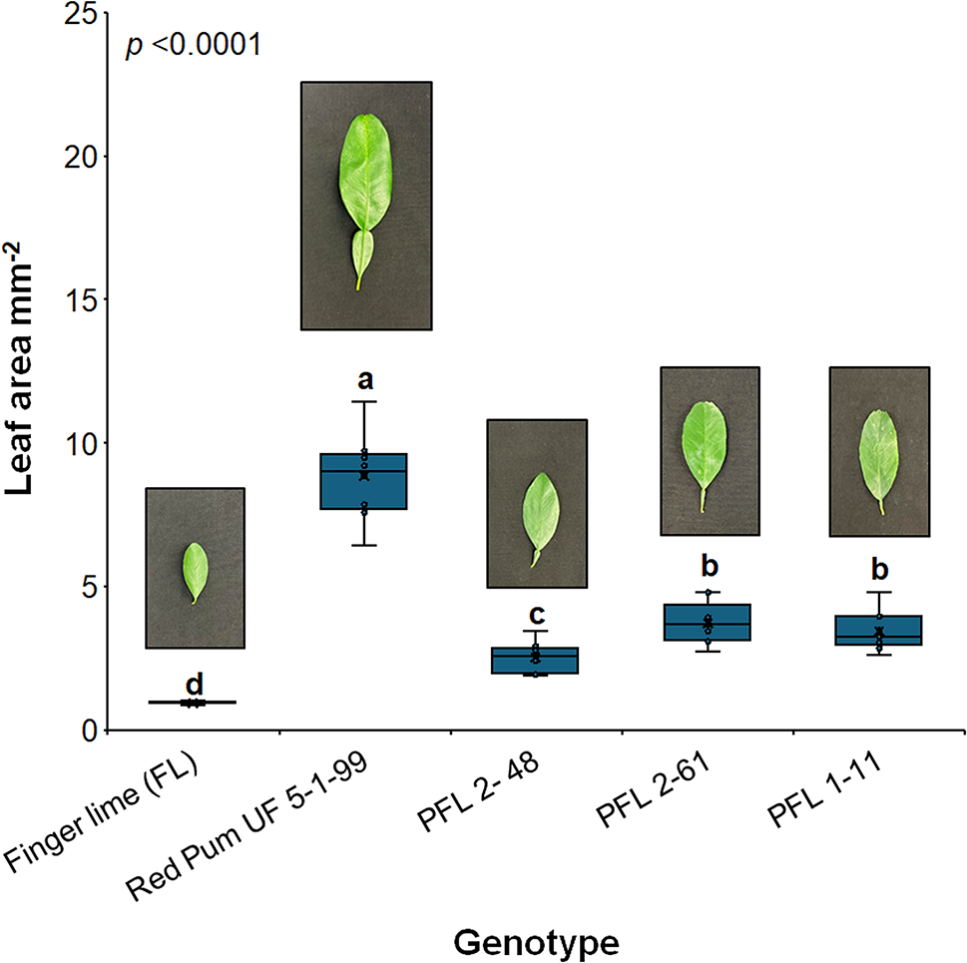
Leaf area comparison between the parents and the hybrids. The box plot represents the leaf areas based on measurements from ten leaves per genotype. The whiskers show the minimum and maximum values, whereas the thick horizontal lines specify the median. Boxes show the interquartile ranges (25th to 75th percentiles of the data), white dots represent the raw data (*n* = 10), and the presented *p* value was determined via one-way ANOVA. ANOVA was followed by Tukey’s honestly significant difference (Tukey HSD) test; thus, different letters indicate statistically significant differences among treatments according to the statistical significance established at *p* < 0.05. The photos on the tops of the boxes show the shapes of the leaves of two-year-old trees cultivated in the greenhouse. The scale indicated in the image corresponds to 20 mm

In this study, we selected an HLB-tolerant finger lime clone as the male parent and a red pulp pummelo (Red Pum UF 5-1-99-2) as the female parent. A large population of the F1 hybrids were clonally propagated on Kuharske rootstock and evaluated in the field under endemic HLB conditions. We selected this specific pummelo as a parent because it grows vigorously and is well-adapted to Florida’s climate. Three hybrid lines (PFL 2–48, PFL 2–61, and PFL 1–11) were utilized to explore the phenotypic variations between hybrids and their respective parents (Red Pum UF 5-1-99-2 and the finger lime) (Fig. 1). Compared with those of the parental pummelo and finger lime, the hybrid leaves exhibited intermediate morphologies. The leaves of the hybrids had small petioles inherited from the pummelo parent. There was no significant difference between PFL 2–61 and PFL 1–11 in terms of the recorded leaf area (3.71 and 3.46 mm^2^, respectively). Compared with the other two hybrids, PFL 2–48 had a lower leaf area.

### Finger lime hybrids exhibit variable responses to *Ca*Las infection

**Fig. 2:**
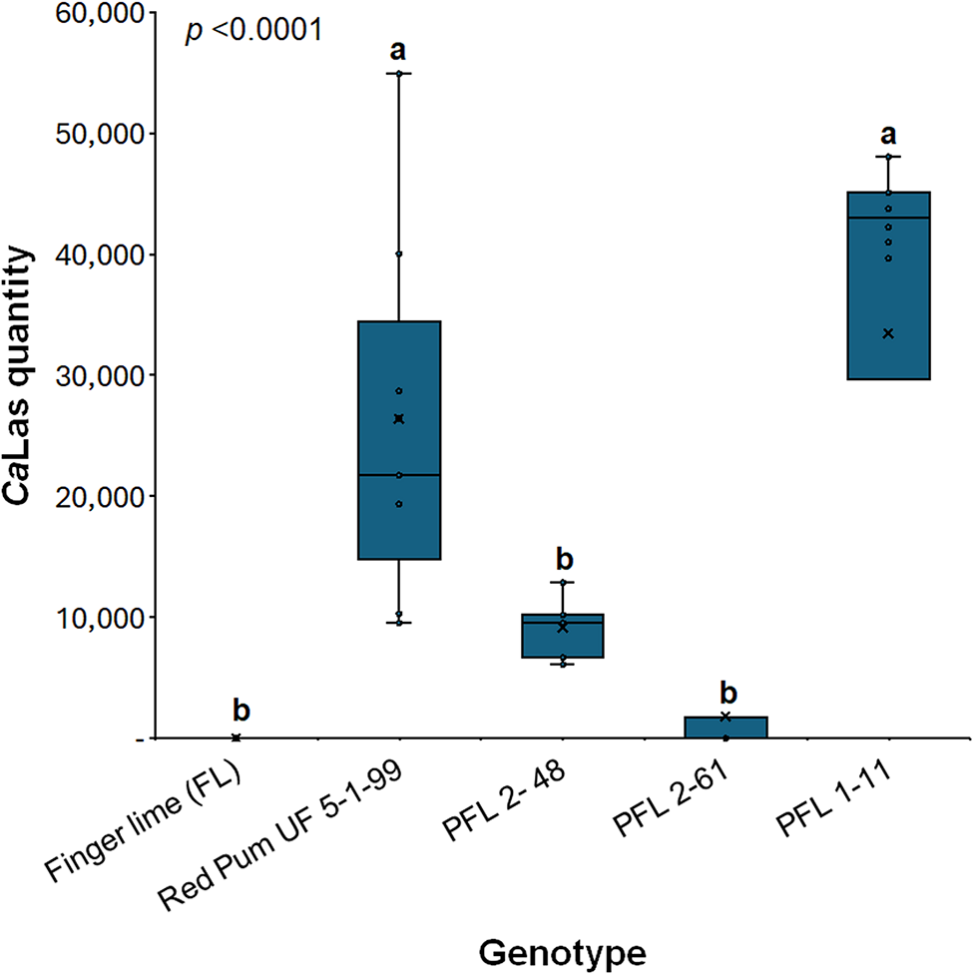
Quantity of *Ca*Las bacterial titers following qPCR of leaf petioles of the Pummelo cultivar (Red Pum UF 5-1-99-2), finger lime tree, and three hybrid varieties. The trees represent propagated trees obtained through budwood stick grafting. These budwoods were collected from field-grown trees naturally infected with *Ca*Las through ACP feeding and were subsequently maintained in a greenhouse for 24 months.The box plot represents the *Ca*Las bacterial titer 10 samples per genotype with 100 mg of fresh midrib tissues used for each sample. The *Ca*Las quantity represents the number of cells per 1 µl of extracted total genomic DNA standardized to a concentration of 25 ng/µl. The whiskers show the minimum and maximum values, whereas the thick horizontal lines specify the median. Boxes show the interquartile ranges (25th to 75th percentiles of the data), black dots represent the raw data (n = 10), and the presented *p*value was determined via one-way ANOVA followed by the Tukey HSD; thus, different letters indicate statistically significant differences among treatments according to the statistical significance established at *p* < 0.05

Finger lime hybrids exhibit anticipated variability in tolerance among seedling-derived populations because of their monoembryonic nature. To study the *Ca*Las infection levels in finger lime, pummelo, and hybrid plants, trees were replicated by stick grafting onto swingle citrimelo and maintained in the greenhouse. Leaf samples were collected two years after propagation and used for further analysis. Finger lime trees exhibited a negative HLB status (undetermined cycle threshold (Ct)) or had a low quantity of *Ca*Las. On the other hand, pummelo trees tested positive for HLB. Compared with the finger lime trees, two of the hybrids (PFL 2–48 and PFL 2–61) showed no significant differences. However, PFL 1–11 exhibited a high level of *Ca*Las (Fig. 2).

### Leaf chlorophyll content, total phenolic compound (TPC) content, and free radical scavenging activity

**Fig. 3:**
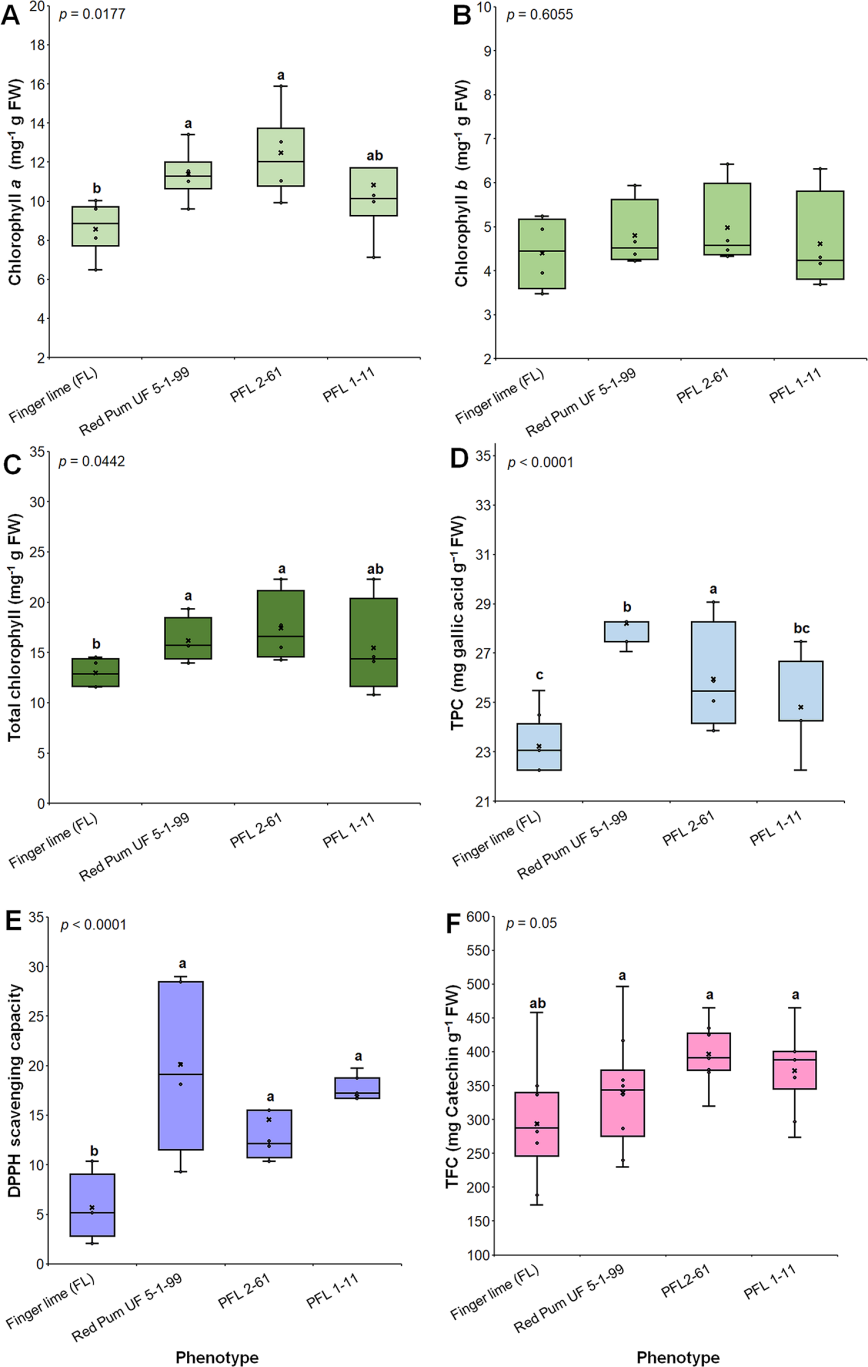
Foliar CHL *a *(A), CHL *b* (B), T CHL content (C), TPC content (D), free radical scavenging activity (E), and total flavonoid content (F) in pummelo hybrids. The box plot represents the level of chemical substances per gram of fresh weight. The whiskers represent the minimum and maximum values, whereas the thick horizontal lines specify the median. Boxes show the interquartile ranges (25^th^ to 75^th^ percentiles of the data), black dots represent the raw data (*n* = 10), and the presented *p *value is based on one-way ANOVA. Different letters indicate statistically significant differences among treatments according to the Tukey HSD test (*p*< 0.05)

Significant variance analysis of the foliar chlorophyll content among the hybrids was implemented via one-way ANOVA. In general, the chlorophyll *a* (CHL *a*), and total chlorophyll (T CHL) contents significantly differed (p values = 0.0177 and 0.0442, respectively). The chlorophyll *b* (CHL *b*) content did not significantly differ between the hybrids and the parents. Compared with those of the other trees, the foliar CHL *a* and T CHL contents of the samples collected from PFL 2–61 were 12.00 and 17 mg g^− 1^ FW, respectively. The finger lime samples had the lowest CHL content (11.52 mg g^− 1^ FW). The foliar pigment content varied between the two hybrids but was not significantly different (Fig. 3).

The TPC content was determined in pummelo hybrid leaves to evaluate the induced oxidation response in the cultivars. There was a significant difference in the TPC content between the hybrids and the parents, with a *p* value < 0.0001, and the TPC content in the plants ranged from 22.74 to 27.72 mg gallic acid g^− 1^ FW. The highest TPC content was recorded in Red Pum UF 5-1-99-2. Compared with the other plants, the finger lime plants had the lowest TPC content, with 22.74 mg gallic acid g^− 1^ FW. The TPC content was lower in PFL 1–11 than in PFL 2–61 (24.31 and 25.47, respectively; Fig. 3d**)**. The highest percentage of DPPH scavenging capacity was recorded for the two hybrids; no significant difference was recorded when the two hybrids were compared (Fig. 3e). The DPPH-radical scavenging activity significantly differed (*p* value < 0.0001) between the parents and the hybrids. The hybrids and their parents had comparable TPC contents. The highest TFC content was observed in PFL 2–61 (396.75 mg catechin g⁻¹ FW), followed by PFL 1–11 (372.38 mg catechin g⁻¹ FW) (Fig. 3f).

### Identification and analysis of DEGs via RNA–seq

**Fig. 4:**
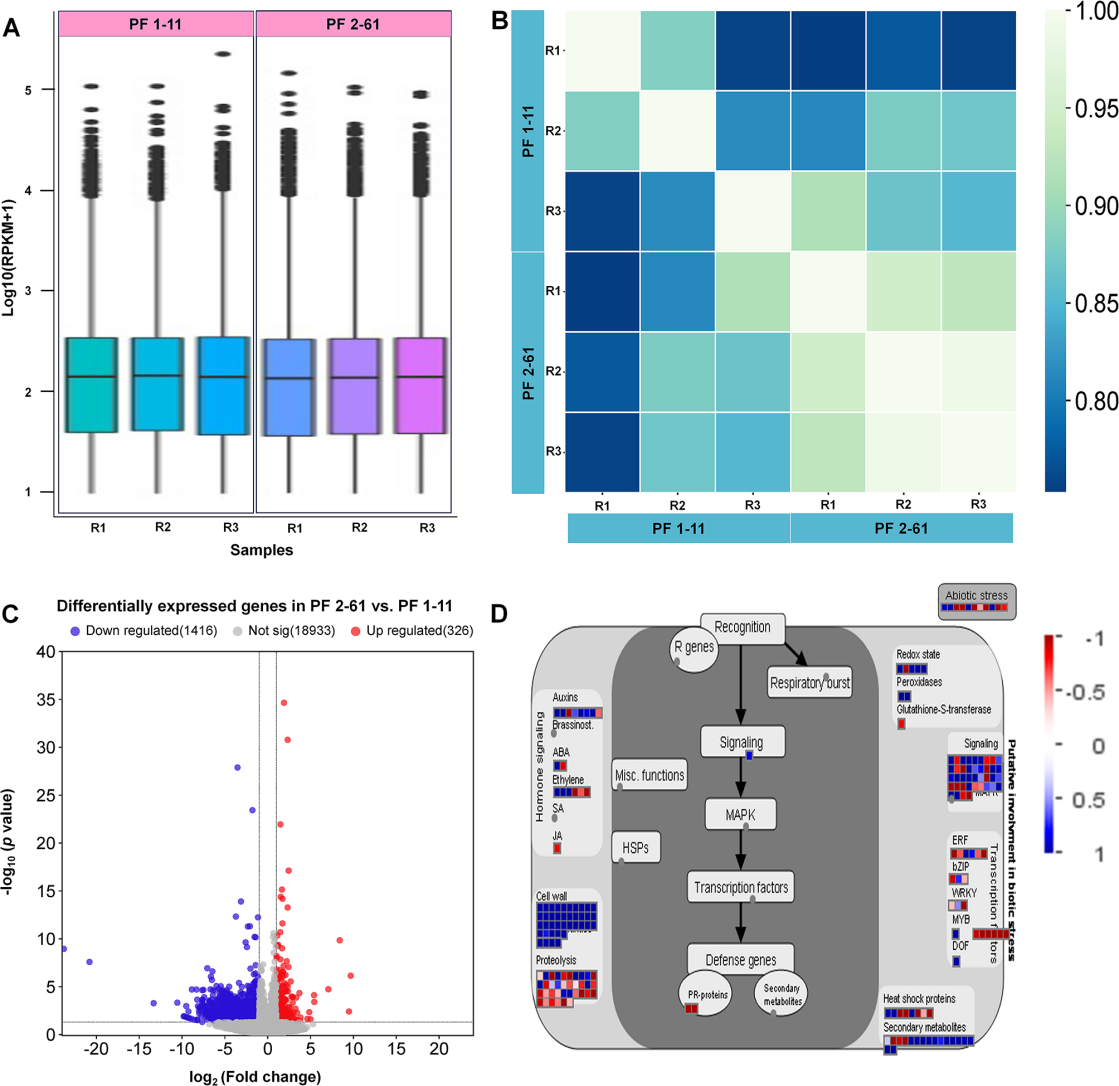
**(**A) Boxplot showing the distribution of gene expression levels in each sample. (B) Heatmap showing the correlation of gene expression between samples.The Pearson correlation coefficients of all gene expressions between each pair of samples were calculated. The correlation coefficients can reflect the similar situation of overall gene expression between each sample. The higher the correlation coefficient is, the more similar the gene expression level is. (C) Volcano plot of upregulated and downregulated DEGs. Genes with an adjusted *p* value of less than 0.05, identified via DESeq, are plotted to illustrate the significance and magnitude of gene expression changes. The plot distinguishes between upregulated (positive log2-fold change) and downregulated (negative log2-fold change) genes. (D) DEGs identified through MAPMAN analysis. This panel provides a visualization of DEGs categorized on the basis of MAPMAN functional annotation. The analysis highlights changes in gene expression across various metabolic and signaling pathways, highlighting the biological relevance of the DEGs

A transcriptome analysis was conducted to interpret the potential biological factors contributing to the variation in HLB tolerance observed in the two hybrids. The resulting raw reads averaged 24,097,444 and 24,051,572 for the PFL 1–11 and PFL 2–61 replicates, respectively (Table 1), with a 100% clean read ratio. These cleaned reads were then mapped onto the *C. clementina* genome. Subsequent analysis of the mapped reads involved differential expression analysis, filtering DEGs on the basis of a|log2 (fold-change)| < 1 and an adjusted *p* value (FDR) ≥ 0.05. Among the 21,532 DEGs identified, 1,416 were downregulated in PFL 2–61 compared with PFL 1–11, whereas 326 were upregulated (Fig. 4 and Table S4). A total of 614 DEGs were mapped via MAPMAN to showcase the biological relevance of the DEGs (Fig. 4b).

**Table 1:** Summary of sequencing, cleaning, and mapping of reads following sequencing of the pummelo x finger lime hybrid samples.^a^

Sample Name	Clean Reads	Clean Base	Mapping ratio	Q20(%)^a^	GC(%)^b^
RF 1-11-1	24,145,196	7,243,558,800	73.39	97.13	44.75
RF 1-11-2	24,136,336	7,240,900,800	72.43	97.39	44.55
RF 1-11-3	24,010,801	7,203,240,300	72.01	97.39	44.57
Average	24,097,444	7,229,233,300	73	97	45
RF 2-61-1	24,002,435	7,200,730,500	70.71	97.63	44.4
RF 2-61-2	24,018,214	7,205,464,200	71.29	97.38	44.51
RF 2-61-3	24,134,068	7,240,220,400	71.05	97.32	44.32
Average	24,051,572	7,215,471,700	71	97	44

Q20(%): Proportion of Q20; ^b^GC(%): Proportion of GC

### Functional classification by GO analysis

**Fig. 5:**
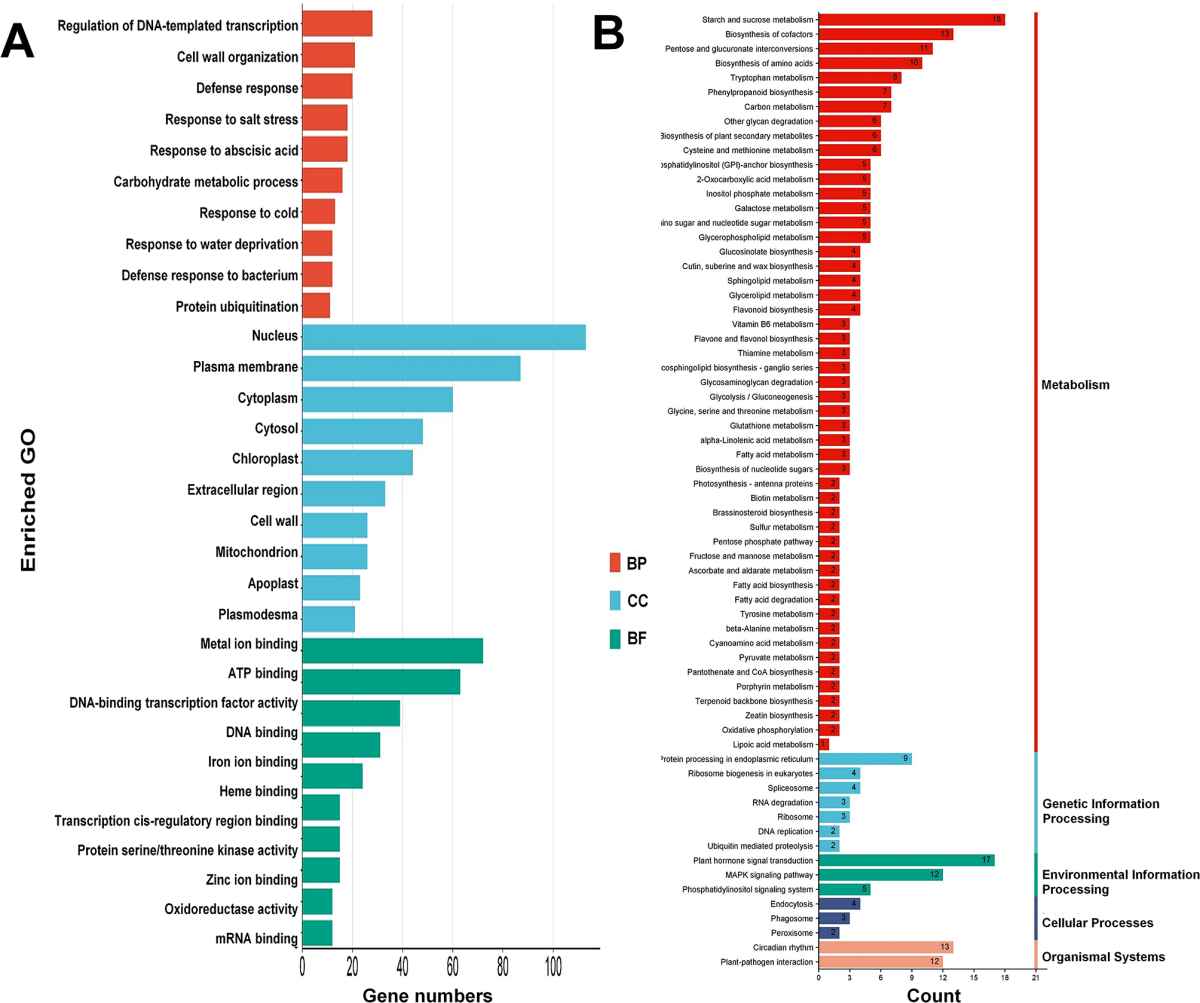
(A) Comparative GO classifications of commonly expressed functionally annotated DEGs from two finger lime hybrid transcriptomes. The genes corresponded to three main categories, cellular component (CC), molecular function (BF), and biological process (BP). (B) Kyoto Encyclopedia of Genes and Genomes (KEGG) pathway mapping of commonly expressed functionally annotated DEGs from two the finger lime hybrid transcriptomes

Gene Ontology (GO) analysis revealed that 614 functionally annotated significant DEGs with coding regions were associated with at least one functional group (Table S3). The 614 DEGs were assigned to three main categories: molecular function (terms, 376), biological process (terms, 521), and cellular component (terms, 119). In the biological process category, the predominant terms were regulated by DNA-templated transcription, cell wall organization, defense response, response to stimulus, and carbohydrate metabolism. In the cellular component category, the nucleus, plasma membrane, cytoplasm, cytosol, chloroplast, and extracellular region were the predominant terms. In the molecular function category, metal ion binding followed by ATP binding, DNA-binding transcription factor activity, DNA binding, iron ion binding, transcription cis-regulatory region binding, and protein serine/threonine kinase activity were the predominant terms (Fig. 5a).

### KEGG pathway mapping

KEGG pathway enrichment analysis revealed the involvement of major pathways. Various metabolic pathways, including 7 involved in genetic information processing, 3 involved in environmental information processing, 3 involved in cellular processes, and 2 involved organismal systems within the KEGG database were enriched in 51 DEGs (Fig. 5b). Most KEGG pathways included more than one DEG. The enriched KEGG Orthology terms (ko–term) with the highest levels of gene representation were starch and sucrose metabolism (ko00500, 18 transcripts), biosynthesis of cofactors (ko01240, 13 transcripts), pentose and glucuronate interconversions (ko00040, 11 transcripts), biosynthesis of amino acids (ko01230, 8 transcripts), phenylpropanoid biosynthesis, and carbon metabolism (ko00940 and ko01200, 5 transcripts each). Notably, plant hormone signal transduction (ko04075, 17 transcripts), the MAPK signaling pathway, and plant‒pathogen interactions (ko04016 and ko04626, 12 transcripts each) were enriched in 17 DEGs.

### Functional differences between tolerant and susceptible finger lime hybrids

Transcriptome analysis revealed variation in the DEGs expressed in both hybrids. The functional proteins associated with those expressed DEGs might regulate the response to *Ca*Las infection or the variation in both hybrid genetics. DEG analysis revealed that the characterized DEGs belonged to one of the following categories: redox homeostasis, abiotic stress, proteolysis, R genes, secondary metabolites, or cell wall-related genes.

### Biotic stress signaling

Differential expression analysis between PFL 2–61 and PFL 1–11 revealed several genes that were significantly upregulated in PFL 2–61. Among them are genes encoding leucine-rich repeat (LRR) domain-containing proteins, which are involved in recognizing pathogens and initiating defense responses. Additionally, members of the cytochrome P450 (CYP2) subfamily had elevated expression levels. We recorded a greater fold change in Abieta-7,13-dien-18-ol hydroxylase/CYP720B1 in PFL 2–61 than in PFL 1–11. Furthermore, we recorded the upregulation of pathogenesis-related proteins (NTF2-like and Bet v I-type allergens), trypsin and protease inhibitors (Kunitz_legume), and germin protein subfamily 1 members 10 and 13 (GLP1-10 and GLP1-13) in PFL 2–61 compared with PFL 1–11. Conversely, genes related to defense mechanisms, including pathogenesis-related thaumatin family protein (PR5) (Thaumatin-like protein 1 precursor - TLP1_PRUPE) and MLO13 - Mildew resistance locus O13 (Table 2), were downregulated.

**Table 2:** Differential gene expression in biotic stress signaling of finger lime hybrids (PFL 2–61 and PFL 1–11)

Gene ID	Log2fc*	*p* value		Main Category
**Pathogen recognition and activation of defense responses**				
Ciclev10007334m	1.3	0.032	Leucine Rich Repeat (LRR) domain-containing protein	Leucine Rich Repeat (LRR) domain-containing protein
Ciclev10003965m	1.8	0.017		LRR_1/LRR_8
Ciclev10030176m	1.7	0.037		LRR_1/LRRNT_2LRR_8
Ciclev10033608m	1.6	0.036		LRR_1/LRR_8
Ciclev10011171m	0.9	0.003		Leucine Rich Repeat/Leucine-rich repeat
Ciclev10025175m	1.4	0.048		Leucine Rich Repeat/Protein tyrosine kinase/Leucine-rich repeat
Ciclev10004039m	1.9	0.008		LRRNT_2)//Leucine rich repeat (LRR_8)
Ciclev10021170m	1.4	0.001	MAPK	Mitogen-Activated Protein Kinase 7
**Synthesis of defense-related compounds and detoxification pathways**				
Ciclev10026966m	5.0	0.025	Cytochrome P450 CYP2 subfamily	Abieta-7,13-dien-18-ol Hydroxylase/CYP720B1
Ciclev10023966m	1.5	0.038		Cytochrome P450 71B21-RELATED
Ciclev10004104m	1.4	0.000		Cytochrome P450 71B21-Related
Ciclev10024587m	1.8	0.043		Cytochrome P450 71B21-Related
Ciclev10031237m	2.2	0.000		Cytochrome P450 78A5-Related
Ciclev10019727m	2	0.000		Cytochrome P450 CYP2 subfamily
Ciclev10000844m	1.9	0.006		Cytochrome P450 CYP2 subfamily
Ciclev10025382m	1.8	0.003		Cytochrome P450 CYP2 subfamily
Ciclev10024390m	1.5	0.025		Cytochrome P450 CYP2 subfamily
Ciclev10009866m	1.4	0.000		Cytochrome P450 CYP2 subfamily
Ciclev10000809m	1.4	0.002		Cytochrome P450 CYP2 subfamily
**Pathogenesis-related proteins**				
Ciclev10002326m	1.3	0.018	NTF2-like domain	Pathogen-related defense protein (NTF2-like)
Ciclev10006074m	1.5	0.024	Bet v I family	Pathogenesis-related protein
Ciclev10006096m	0.9	0.044		Pathogenesis-related protein Bet v I family (Bet_v_1)
Ciclev10012489m	-5.9	0.0003	Thaumatin family	PR-5
Ciclev10033952m	-1.3	0.0003		TLP1_PRUPE
Ciclev10000933m	-3.5	0.001	Mlo family	Mildew resistance locus O13 (MLO13)
Ciclev10022211m	4.6	0.022	Regulation of protease activity	Trypsin and Protease Inhibitor
Ciclev10002395m	2.1	0.015		Trypsin and protease inhibitor
Ciclev10022911m	5.4	0.000		Trypsin and Protease
Ciclev10022906m	3.8	0.017		Potato Inhibitor I Family
Ciclev10022111m	2.7	0.007	Germin-like proteins (GLPs)	GLP1-10
Ciclev10022118m	4.8	0.005		GLP1-13
Ciclev10033943m	-6.2	0.004		GPL1-1
Ciclev10022159m	-5.1	0.027		GPL2

*Negative values represent downregulation in PFL 2–61 and upregulation in PFL 1–11

### Redox homeostasis

Some identified DEGs in PFL 2–61 compared with PFL 1–11 are involved in regulating key genes associated with redox balance and defense against biotic stress. Three redox-related genes were downregulated: ciclev10019057m (TTL1 - Tetratricopetide-repeat thioredoxin-Like 1), ciclev10032233m (FSD2 - Fe superoxide dismutase 2), and ciclev10026205m (APX3 - Ascorbate peroxidase 3); however, we recorded the upregulation of thioredoxin H-type 7, peroxidases and lactoperoxidases in PFL 2–61. Furthermore, we recorded the upregulation of oxidoreductases belonging to the 2OG-FE II oxygenase family and the 5 kDa heat shock protein localized in mitochondria.

### Secondary metabolism

**Fig. 6:**
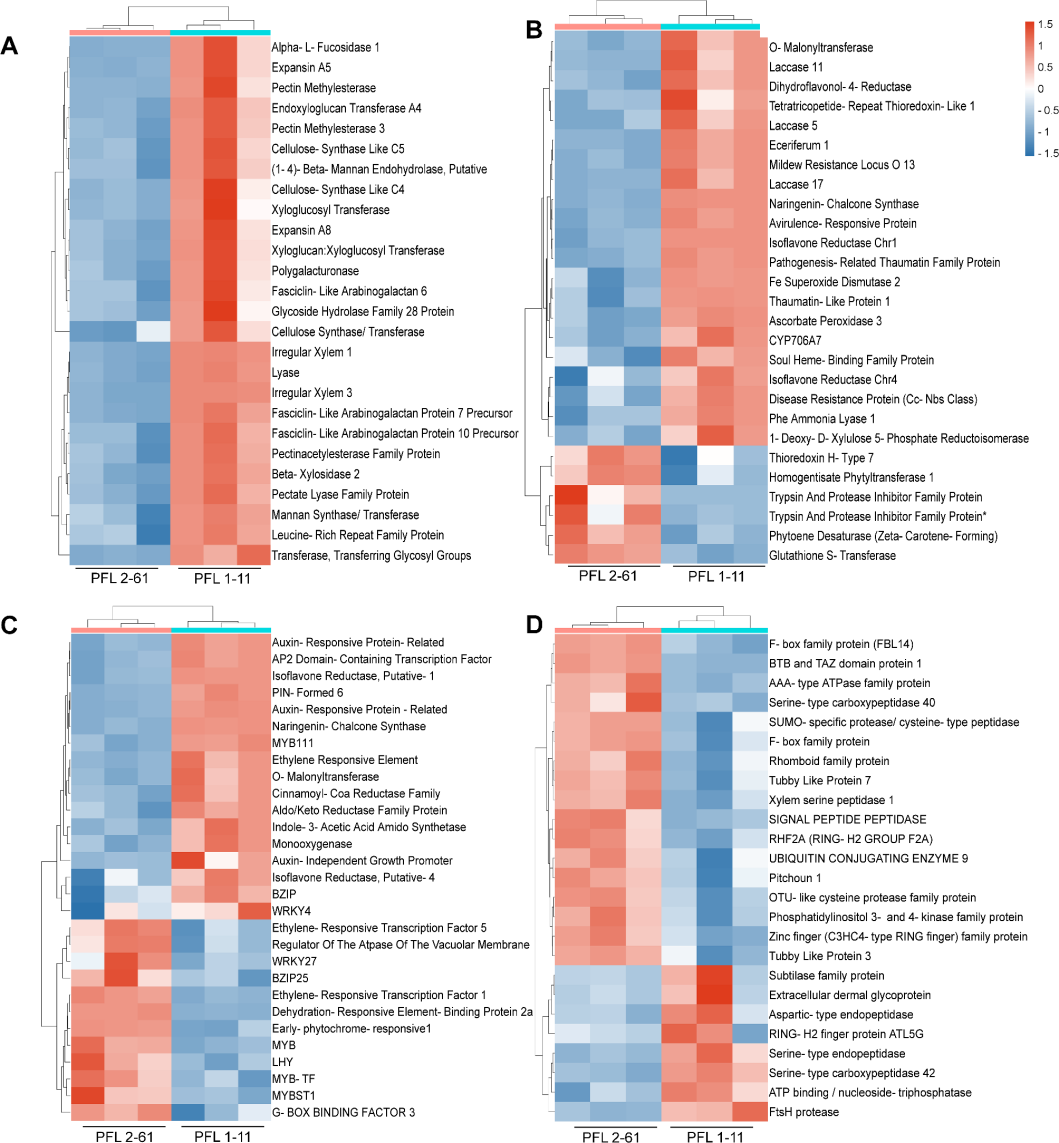
Two-way hierarchical cluster analysis (HCA) associated with a heatmap of functionally characterized DEGs related to the cell wall (**A**), DEGs related to redox homeostasis and biotic stress signaling (**B**), DEGs related to phytohormones (**C**), and classes of proteases and ubiquitin-related proteins (**D**) for two hybrids, PFL 2–61 and PFL 1–11. The orange color denotes upregulated DEGs, and the blue color denotes downregulated DEGs. The gradation from blue to gray represents the transition from large to small values of FPKM-normalized log^2^-transformed counts. Rows correspond to individual transcripts, whereas columns correspond to three repeats for each hybrid. Low numerical values are blue, whereas high numerical values are red (the scale is presented at the top right corner of the heatmap)

The RNA-seq data revealed significant changes in the expression of genes associated with various metabolic pathways, including phenylpropanoid, isoprenoid, simple phenol, and flavonoid metabolic pathways, in PFL 2–61 compared with those in PFL 1–11. The expression of the gene ciclev10011175m, identified as phenylalanine ammonia lyase 1 (PAL1), which encodes phenylalanine ammonia lyase, was downregulated. This enzyme is crucial in the phenylpropanoid pathway, leading to the biosynthesis of various secondary metabolites, including flavonoids and lignin. Among the isoprenoid metabolism-related genes, ciclev10020004m (DXR-1-deoxy-D-xylulose 5-phosphate reductoisomerase) was downregulated in PFL 2–61 compared with PFL 1–11. In the context of secondary metabolism, including simple phenol metabolism, several laccase genes, including ciclev10031070m (LAC5), ciclev10025287m (LAC11), ciclev10028036m, ciclev10011362m and ciclev10028090m (LAC17), were downregulated in PFL 2–61. Laccases play a role in the oxidation of phenolic compounds and are involved in lignin biosynthesis. However, upregulation was observed in ciclev10003300m (HPT1 - Homogentisate Phytyltransferase 1) in PFL 2–61. HPT1 is involved in the phytoene dehydrogenase pathway. Two transferase family proteins (ciclev10028457m and ciclev10015234m) related to phenylpropanoid metabolism were upregulated in PFL 2–61 compared with PFL 1–11 (Fig. 6b). Some DEGs, such as Flavonoid 3’-monooxygenases and flavonoid 3’-hydroxylases, are directly involved in flavonoid biosynthesis. Moreover, genes associated with flavonoid metabolism, including flavin-binding monooxygenase-like (FMO-like) and flavonoid 3’-monooxygenase/flavonoid 3’-hydroxylase, and five genes associated with tetrahydroberberine oxidase/THB oxidase biosynthesis are also upregulated in PFL 2–61. The gene ciclev10020021m, annotated as an anthocyanin 5-aromatic acyltransferase, was downregulated in PFL 2–61 compared with PFL 1–11. Additionally, genes associated with the flavonoid and anthocyanin biosynthesis pathways, including *5MAT* (O-malonyltransferase), *CHS* (naringenin-chalcone synthase 2), cinnamoyl-CoA reductase, and *DFR* (dihydroflavonol-4-reductase) family member ciclev10003684m, had decreased expression in PFL 2–61 compared with PFL 1–11. Furthermore, other enzymes involved in the flavonoid pathway, such as monooxygenase (ciclev10006920m or CYP706A7) and two isoflavone reductases (ciclev10001946m and ciclev10028904m), were also downregulated in PFL 2–61.

### Cell wall-related DEGs

All the cell wall-related transcription factors were downregulated in PFL 2–61 compared with PFL 1–11. These DEGs included cellulose synthesis genes such as cellulose synthase-like C5 (*CSLC05*) and *CESA7*/*IRX3*, along with those involved in hemicellulose synthesis, such as *PGSIP3*. Cell wall proteins, including fasciclin-like arabinogalactan proteins (*FLA6*, *FLA10*, and *FLA12*), were identified. Additionally, we reported the downregulation of genes related to cell wall structure and signaling, including *RPK1_IPONI*, a leucine-rich repeat family protein. Enzymes such as *BXL2*, *ATFUC1*, and a putative (1–4)-beta-mannan endohydrolase were also identified. Pectin degradation involves a spectrum of enzymes, including the glycoside hydrolase family, pectate lyases, and polygalacturonases. Genes such as EXGT-A4, EXP8, EXPA5, pectin esterase (PME), and acetyl esterase further elucidate the complexity of pectin and cell wall modifications. Furthermore, a wax metabolism-related gene, *CER1*-Eceriferum 1, was downregulated in PFL 2–61, indicating potential changes in the composition of cuticular waxes in response to *Ca*Las infection (Fig. 6a).

### **Phytohormone-related factors**

The investigation of phytohormone-related gene expression in finger lime hybrids revealed significant alterations under *Ca*Las infection. The synthesis-related gene ciclev10014639m, associated with abscisic acid (ABA) production through 9-cis-epoxy carotenoid dioxygenase, was markedly downregulated in PFL 2–61; however, ABA-responsive element-binding factor 3 (*ABF3*) was upregulated. Six auxin-responsive transcription factors, including *PIN6* (ciclev10000794m), *DFL1* (ciclev10007746m), and various auxin-related proteins, were downregulated in PFL 2–61. However, the auxin-induced growth promoter *AILP1* (ciclev102005766m) was upregulated. Within the AP2/EREBP family, which is implicated in transcriptional regulation, ciclev10009447m (*SHN1*) and ciclev10009827m (AP2 domain-containing transcription factor) were downregulated in PFL 2–61. Conversely, ciclev10015144m (ERF1_ORYSA), ciclev10005750m (AP2 domain-containing transcription factor), ciclev10032029m (dehydration-responsive element-binding protein 2 A), and ciclev10031846m (*RAV2*) were upregulated. Jasmonate signaling, crucial for stress responses, revealed the upregulation of ciclev10014199m (LOX2), a lipoxygenase involved in jasmonate synthesis, in PFL 2–61 compared with that in PFL 1–11 (Fig. 6c).

Transcription factor expression analysis revealed significant changes in the regulation of key transcription factor families, as upregulation of several MYB-related transcription factors, e.g., early-phytochrome-responsive 1 (EPR1), and late elongated hypocotyl (LHY), was observed. Additionally, members of the MYB-related transcription factor family, ciclev10032167m, ciclev10012263m, and ciclev10005411m, were also upregulated in PFL 2–61 compared with PFL1-11. However, *MYB111* (myb domain protein 111) was downregulated in PFL 2–61. Within the WRKY domain transcription factor family, *WRKY27* (ciclev10005095m) was downregulated, whereas ciclev10031225m (*WRKY4*) was upregulated in PFL 2–61. In the *bZIP* transcription factor family, ciclev10020314m (*GBF3*) and ciclev10020133m (*BZIP25*) were upregulated, whereas ciclev10028062m (bZIP_1) was downregulated in PFL 2–61. Among the AP2/EREBP family members, ciclev10009447m (*SHN1*) and ciclev10009827m (AP2 domain-containing transcription factor, putative) were downregulated in PFL 2–61 (Fig. 6c).

Furthermore, we recorded variation in the expression of genes encoding various classes of proteases and ubiquitin-related proteins in PFL 2–61 compared with PFL 1–11. Eighteen transcription factors encoding protein degradation subtilases, including *subtilisin serine protease-related 3* (ciclev10007470m), *AIR3* (ciclev10014355m), ASP1_ORYSA (ciclev10001122m), two aspartate proteases (serine carboxypeptidase-like 42 ciclev10000998m), metalloprotease, FtsH protease (ciclev10018718m) and eleven ubiquitin E3 RING zinc finger family proteins. Conversely, ciclev10015833m (aspartic-type endopeptidase), ciclev10020105m (SUMO-specific protease/cysteine-type peptidase), ciclev10032840m (OTU-like cysteine protease family protein), ciclev10011658m (serine protease), ciclev10031327m (serine carboxypeptidase-like 40), ciclev10007229m (AAA type - expressed RPT1 SpoVK PRK03992 AAA HflB COG1223 hflB), and ciclev10030931m and ciclev10011271m (expressing phosphatidylinositol 3- and 4-kinase family protein - PI3 and PI4 kinase), and ciclev10013033m (ubiquitin E2 - UBC9) were upregulated in PFL 2–61 compared with PFL 1–11. Furthermore, three ubiquitin E3 SCF FBOXs (ciclev10005501m, ciclev10025548m, ciclev10005517m, ciclev10020367m) and one ubiquitin E3 BTB/POZ Cullin3 BTB/POZ (ciclev10020762m) was upregulated in PFL 2–61 compared with PFL 1–11 (Fig. 6d).

### Sieve element occlusion N-terminus (SEO_N) genes are downregulated in PFL 2–61

SEO_N genes are crucial for the maintenance of phloem integrity because they facilitate sieve element occlusion through callose deposition. In the PFL 2–61 hybrid, which is more tolerant to biotic stress than PFL 1–11, we observed a downregulation of several transcripts related to the Sieve Element Occlusion N-Terminus (SEO_N). These genes included key genes such as Ciclev10015478m, Ciclev10019109m, Ciclev10030843m, and Ciclev10014015m (Table 3).

**Table 3:** Differential gene expression of phloem-related genes in finger lime hybrids (PFL 2–61 and PFL 1–11)

Gene name	Log2fc*	*p* value	Function
Ciclev10015478m	-3.52	0.0007	Callose synthase 3
Ciclev10019109m	-2.04	0.0211	Sieve element occlusion N-terminus (SEO_N)
Ciclev10030843m	-3.21	0.0006	
Ciclev10014015m	-1.41	0.0270	

*Negative values represent downregulation in PFL 2–61 and upregulation in PFL 1–11

### qPCR validated the RNA-seq data

**Fig. 7:**
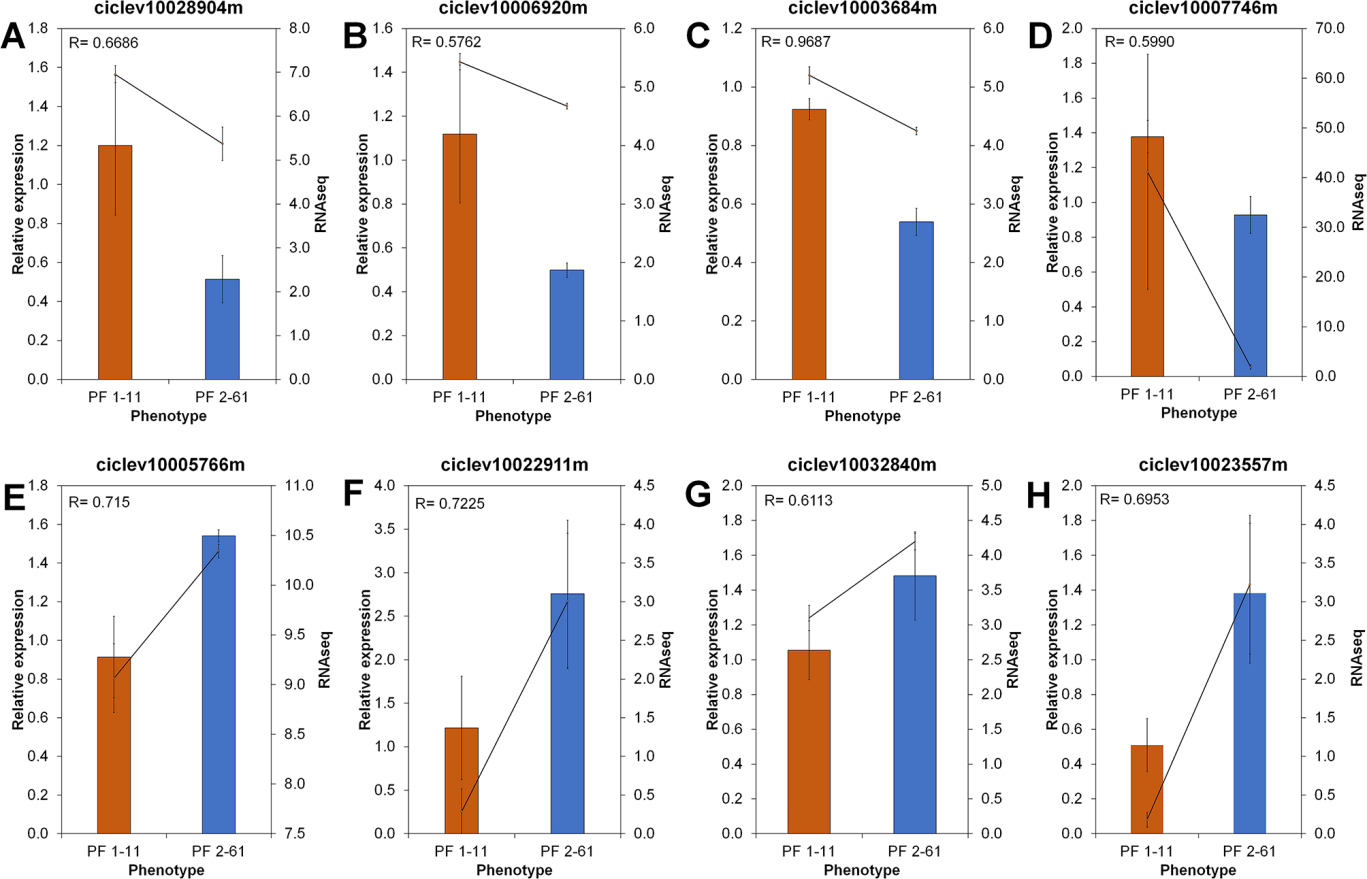
Verification of the expression levels of selected downregulated (**A**-**D**) or upregulated (**E**-**H**) DEGs in the finger lime hybrid (PFL 2–61) compared with PFL 1–11, as determined by qPCR (2^−ΔΔCt^). A Pearson correlation coefficient (r) greater than 0.5 is considered positive and strong (*n* = 3)

**Fig. 8:**
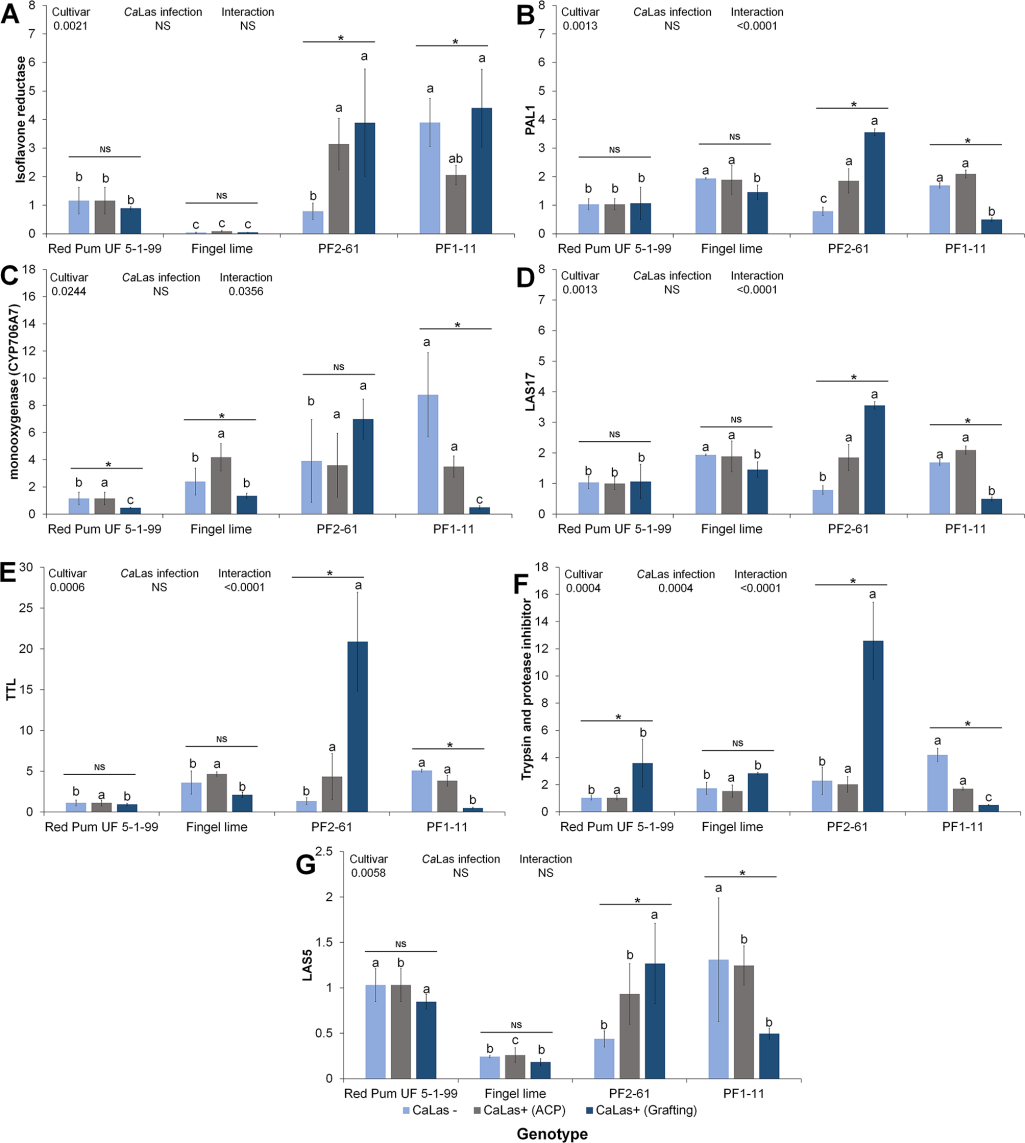
The relative transcript levels of some selected genes in response to *Ca*Las infection were calculated via real-time PCR. *Ca*Las infection can be induced by ACP feeding or grafting with infected plant materials. The *Ca*Las-free (control) and infected trees were maintained separately in a screen house. The control trees were confirmed to be negative for *Ca*Las before further comparison. Different letters indicate statistically significant differences among treatments for each genotype according to the Tukey HSD test (*p* < 0.05). Asterisks above the standard error bars indicate statistically significant differences, whereas “ns” signifies no significant difference at the 5% level (ANOVA, Tukey’s test). The data are presented as the means ± SEs (*n* = 3)

Eight genes were selected for qRT-PCR verification to confirm the reliability of the transcriptome data (Fig. 7). The qPCR results were consistent with the RNA-seq data, and the mRNA expression of these genes was either significantly up- or downregulated in PFL 2–61 compared with PFL 1–11. We also checked the gene expression of some selected genes under inducible *Ca*Las infection to validate our data in different trees. Compared with that in the parents, the expression of the isoflavone reductase gene in the finger lime hybrid leaves was greater. The expression levels of the PAL1, monooxygenase, Las17, TTL, trypsin, and protease inhibitor genes were upregulated following *Ca*Las infection caused by grafting in PFL 2–61. The expression of these genes varied among the tested plants in response to *Ca*Las infection. Finger lime and pummelo plants demonstrated increased trypsin and protease inhibitor levels in response to *Ca*Las infection caused by grafting. Compared with those of the control plants, the response of the finger lime and pummelo plants to *Ca*Las infection slightly differed (Fig. 8).

## Discussion

In the present study, the response of two genetically similar finger lime hybrids to infection with *Ca*Las was evaluated using leaf transcriptome analyses. Several significant DEGs were identified and functionally annotated. GO enrichment analysis revealed that for the biological process category, the GO terms and KEGG pathways corresponding to cell wall organization, defense response to stimulus, carbohydrate metabolism process, and the generation of several precursor metabolites were the most enriched in the DEGs of the finger lime hybrids. Previous transcriptome analyses linked to HLB have highlighted significant influences on GO terms and KEGG pathways associated with stimuli, the MAPK signaling pathway, and metabolic processes [11, 39–42]. Foliar chlorophyll content analysis revealed variation among hybrids. Although variations in the Ct value of *Ca*Las were recorded, the plants remained asymptomatic throughout the two-year study. Compared with PFL 1–11 and the finger lime parent, PFL 2–61 had higher CHL *a* and T CHL levels. The chlorophyll content is indicative of a plant’s photosynthetic capacity and overall health and reflects variations in the physiological responses of different citrus phenotypes. Furthermore, we recorded an increase in the TPC content and free radical scavenging activity in PFL 2–61, indicating differential antioxidant properties.

Plant defense mechanisms involve a complex interplay of various factors, including proteins and enzymes. Among them, members of the CYP2 subfamily, NTF2-like domain, Betv I family, Thaumatin family, Mlo family, Kunitz family, trypsin and protease inhibitors, and Germin-like proteins (GLPs) had differential expression when the transcripts of the two hybrids were compared. Cytochrome P450 enzymes (CYPs) bolster plant defense systems by managing the production of secondary metabolites, increasing the scavenging of reactive oxygen species (ROS), and regulating the levels of phytohormones such as abscisic acid (ABA) and jasmonate [43]. We recorded a greater fold change in Abieta-7,13-dien-18-ol hydroxylase/CYP720B1 in the tolerant hybrid (PFL 2–61) than in the susceptible hybrid (PFL 1–11). The cytochrome P450 enzyme CYP720B1 (PtAO) was found to be essential for the production of diterpene resin acids (DRAs) in loblolly pine by catalyzing multiple oxidation steps on various diterpene and diterpenoid intermediates. Its expression increases in response to simulated insect attack, highlighting its role in conifer defense through the generation of diverse DRA metabolites [44]. The mitogen-activated protein kinase (MAPK/MPK) cascade is a key intracellular signaling pathway that controls plant growth, development, reproduction, and reactions to both biotic and abiotic stresses [45]. We recorded the upregulation of mitogen-activated protein kinase kinase 7-related genes in the tolerant hybrid compared with the susceptible hybrid. We also observed the upregulation of several leucine-rich repeat (LRR) domain-containing proteins, along with protein tyrosine kinase domains, in the two hybrids. These proteins play critical roles in pathogen recognition and signal transduction, contributing to the activation of defense responses [45].

The Kunitz family of trypsin and protease inhibitors inhibits the activity of digestive enzymes in herbivores, thereby reducing nutrient availability for pests [46]. Yu and Killiny [47] reported the presence of proteases and endoglucanases in the saliva of *D. citri*, suggesting a potential connection with the insect’s ability to degrade or inhibit citrus defense proteins. Previously, we reported downregulation of the expression of protease inhibitors genes in HLB-infected ‘Valencia’ sweet orange trees and upregulation in finger lime [12]. Herein, we recorded the upregulation of four cysteine protease inhibitors in the most tolerant PFL 2–61 tree.

Redox homeostasis plays a crucial role in plant defense mechanisms, and alterations in these genes may affect a plant’s ability to mitigate oxidative stress induced by the pathogen [48]. We recorded the upregulation of ascorbate peroxidase 3 (*APX3*), Fe superoxide dismutase 2 (*FSD2*) and tetratricopeptide-repeat thioredoxin-like 1 (*TTL1*) in PFL 1–11; however, we recorded the upregulation of glutathione S-transferase and thioredoxin H-type 7 in PFL 2–61. Among the proteins induced during plant defense, plant peroxidases play roles in the reinforcement of the cell wall, increased production of ROS, and increased production of phytoalexins [49]. The induction of peroxidases has been reported previously in response to *Ca*Las infection in susceptible *C. sinensis* [14]. In the late stage of *Ca*Las infection in Mexican lime, two *SOD* genes, one *CAT* gene, and four *Prx* genes are repressed [40]. The upregulation of redox-related genes such as *APX3*,* FSD2*, and *TTL1* in PFL 1–11 indicates a potential disruption in redox homeostasis in response to *Ca*Las infection. GSTs have been suggested to be important modulators of citrus tolerance to HLB disease [11]. Additionally, we recorded the upregulation of germin-like proteins that contribute to pathogen resistance by increasing ROS production and strengthening cell walls [50].

Unlike many other pathogens, HLB-causing bacteria do not directly interact with the plant cell wall. Therefore, despite the role of the cell wall as a barrier against external threats, it does not directly impede the progression of HLB infection. Instead, bacteria block the phloem, where they disrupt nutrient transport and ultimately cause systemic damage to the plant. In response to *Ca*Las infection, a total of 26 genes related to cell wall metabolism were downregulated in the PFL 2–61 hybrid. The downregulation of a subset of cellulase synthases is in agreement with previous reports [51, 52], where it was suggested that the downregulation of these enzymes could be related to cell wall strengthening and consequently to the tolerance displayed by Kaffir lime (*C. hystrix*) and “Jackson” grapefruit. We also observed the downregulation of pectin methyl esterase (PME). The downregulation of PME enzymes has been observed in the tolerant “Jackson” grapefruit and rough lemon (*C. jambhiri*) cultivars [51, 53]. Pectin de-esterification by pectin methyl esterases (PMEs) renders the cell wall more susceptible to enzymatic degradation. Consequently, alterations in pectin methyl esterification indirectly impact the success of pathogen invasion and disease progression. Similarly, fasciclin-like arabinogalactan proteins (AGPs) were also downregulated in PF 2–61. AGPs play defensive roles against pathogenic infection by secreting and aggregating AGPs at affected sites, limiting pathogen propagation [54]. Additionally, we recorded the upregulation of irregular xylem genes on chromosomes 1 and 3 in the susceptible hybrid. Irregular xylem formation in *Ca*Las-infected trees impairs water movement, resulting in wilting, nutrient limitations, stunted growth, and compromised fruit quality [55, 56].

Secondary metabolites play essential roles in plant defense. Previous studies have shown that the expression of most genes involved in secondary metabolism, including terpenoid, phenylpropanoid, and flavonoid biosynthesis pathways, is mostly induced in *Ca*Las-infected leaves [57]. *PAL*, which functions as the gateway enzyme, directs metabolic flux from the shikimate pathway to various branches of phenylpropanoid metabolism by converting phenylalanine into trans-cinnamic acid. The upregulation of the *PAL1* and laccase genes enhances phenylpropanoid and flavonoid biosynthesis, which serve as defense molecules against pathogens. Compared with PFL 2–61, the expression of flavonoid biosynthesis transcription factors such as dihydrokaempferol 4-reductase, naringenin-chalcone synthase, and isoflavone reductase in PFL 1–11 were increased. In our biochemical assays, we recorded comparable TFC contents in the two hybrids. Moreover, flavonoids have been found to possess antifungal and antioxidant activities [58].

Phytohormones play key roles in regulating various physiological processes, including defense responses against pathogens [59]. We recorded the downregulation of ABA synthesis-related genes and the upregulation of jasmonate-related genes in PFL 2–61. This finding aligns with our previous findings, which reported the upregulation of several candidate genes involved in gibberellic acid (GA) synthesis and the production of bioactive GA in tolerant finger lime *Ca*Las infection [12].

Transcription factor expression analysis revealed significant changes in key regulatory genes, including those in the MYB, WRKY, AP2/EREBP, and bZIP families. The observed changes in their expression suggest the activation of complex regulatory networks involved in the plant’s defense response to *Ca*Las infection. Members of the WRKY family are implicated in regulating the transcriptional reprogramming associated with plant immune responses, and they may act as positive and negative regulators of disease resistance [60]. WRKY upregulation was detected in *Ca*Las-infected susceptible *C. sinensis* and red tangerine; however, no significant deregulation was detected in tolerant rough lemon [53, 61]. These results suggest that WRKY deregulation may not be a differential response between susceptible and tolerant citrus cultivars. Other genes encoding bZIP and C2H2 zinc finger transcription factors were upregulated in *Ca*Las-infected fruits [11]. The regulation of bZIPs in HLB-affected citrus may be associated with reported modifications observed in energy metabolism [62]. Collectively, these proteins form a complex network that enables plants to withstand environmental challenges and defend against pathogens, ensuring plant survival and adaptation in diverse ecological niches.

## Conclusions

The complexity of HLB and its devastating effect on citrus trees requires comprehensive research efforts to understand the mechanisms by which the pathogen provokes the disease and, accordingly, to develop effective mitigation strategies. Despite the challenges created by HLB, promising avenues for resistance or tolerance have emerged, particularly through the utilization of HLB tolerant citrus relatives such as the finger lime. Transcriptome analyses have provided valuable insights into the molecular responses of citrus plants to *Ca*Las infection, highlighting alterations in gene expression related to various biological processes, including cell wall metabolism, defense responses, and carbohydrate metabolism. Our study greatly contributes to ongoing efforts to develop resilient citrus varieties by revealing the molecular mechanisms underlying susceptibility and tolerance.

## Electronic supplementary material

Below is the link to the electronic supplementary material.

Supplementary Material 1

## Data Availability

Data was submitted to NCBI under the BioProject ID: PRJNA1219116.

## Abbreviations

ABA: Abscisic Acid
APX: Ascorbate Peroxidase
*Ca*Las: *Candidatus* Liberibacter asiaticus
CAT: Catalase
CHL: Chlorophyll
CYP: Cytochrome P450
DEGs: Differentially Expressed Genes
FSD: Fe Superoxide Dismutase
GO: Gene Ontology
GST: Glutathione S-Transferase
HLB: Huanglongbing
KEGG: Kyoto Encyclopedia of Genes and Genomes
LRR: Leucine-Rich Repeat
MAPK/MPK: Mitogen-Activated Protein Kinase
MYB: Myeloblastosis Transcription Factor
PAL: Phenylalanine Ammonia Lyase
ROS: Reactive Oxygen Species
SOD: Superoxide Dismutase
TFC: Total Flavonoid Content
TPC: Total Phenolic Content
WRKY: WRKY Transcription Factor
qPCR: Quantitative Polymerase Chain Reaction
ANOVA: Analysis of Variance
DXR: 1-Deoxy-D-Xylulose 5-Phosphate Reductoisomerase
LAC: Laccase
CESA: Cellulose Synthase
CSL: Cellulose Synthase-Like
RPK: Receptor Protein Kinase

## References

[1] Liu YQ, Heying E, Tanumihardjo SA. History, global distribution, and nutritional importance of citrus fruits. Compr Rev Food Sci Food Saf. 2012;11:530–45.

[2] Bové JM. Huanglongbing: a destructive, newly-emerging, century-old disease of citrus. J Plant Pathol. 2006;88:7–37.

[3] Brlansky R, Rogers M. Citrus huanglongbing: understanding the vector-pathogen interaction for disease management. Plant Health Progr. 2007;10. 10.1094/APSnetFeature-2007-1207.

[4] Wang N, Pierson EA, Setubal JC, Xu J, Levy JG, Zhang Y, et al. The candidatus liberibacter–host interface: insights into pathogenesis mechanisms and disease control. Annu Rev Phytopathol. 2017;55:451–82. 28637377 10.1146/annurev-phyto-080516-035513

[5] Killiny N. Made for each other: vector–pathogen interfaces in the huanglongbing pathosystem. Phytopathology. 2022;112:26–43. 34096774 10.1094/PHYTO-05-21-0182-FI

[6] Mahmoud LM, Weber KR, Trama T, England G, Dutt M. A comparative study between ‘Parson Brown’and ‘Hamlin’Sweet oranges growing under endemic huanglongbing conditions in Florida. HortScience. 2023;58:1149–60.

[7] Milne AE, Teiken C, Deledalle F, van den Bosch F, Gottwald T, McRoberts N. Growers’ risk perception and trust in control options for huanglongbing citrus-disease in Florida and California. Crop Prot. 2018;114:177–86.

[8] Graham J, Gottwald T, Setamou M. Status of huanglongbing (HLB) outbreaks in Florida, California and Texas. Trop Plant Pathol. 2020;45:265–78.

[9] Grosser J, Gmitter F, Castle W. Breeding citrus rootstocks to mitigate Huanglongbing (HLB, or citrus greening disease). Acta Hortic. 2014;117:83–8.

[10] Dutt M, Mahmoud LM, Chamusco K, Stanton D, Chase CD, Nielsen E, et al. Utilization of somatic fusion techniques for the development of HLB tolerant breeding resources employing the Australian finger lime (Citrus australasica). PLoS ONE. 2021;16:e0255842. 34375348 10.1371/journal.pone.0255842 PMC8354479

[11] Martinelli F, Uratsu SL, Albrecht U, Reagan RL, Phu ML, Britton M, et al. Transcriptome profiling of citrus fruit response to huanglongbing disease. PLoS ONE. 2012;7:e38039. 22675433 10.1371/journal.pone.0038039 PMC3364978

[12] Weber KC, Mahmoud LM, Stanton D, Welker S, Qiu W, Grosser JW, et al. Insights into the mechanism of Huanglongbing tolerance in the Australian finger lime (Citrus australasica). Front Plant Sci. 2022;13:1019295. 36340410 10.3389/fpls.2022.1019295 PMC9634478

[13] Killiny N, Hijaz F. Amino acids implicated in plant defense are higher in Candidatus Liberibacter asiaticus-tolerant citrus varieties. Plant Signal Behav. 2016;11:e1171449. 27057814 10.1080/15592324.2016.1171449 PMC4883877

[14] Albrecht U, Bowman KD. Gene expression in Citrus sinensis (L.) Osbeck following infection with the bacterial pathogen Candidatus Liberibacter asiaticus causing Huanglongbing in Florida. Plant Sci. 2008;175:291–306.

[15] Folimonova SY, Robertson CJ, Garnsey SM, Gowda S, Dawson WO. Examination of the responses of different genotypes of citrus to huanglongbing (citrus greening) under different conditions. Phytopathology. 2009;99:1346–54. 19900000 10.1094/PHYTO-99-12-1346

[16] Killiny N, Jones SE, Hijaz F, Kishk A, Santos-Ortega Y, Nehela Y, et al. Metabolic profiling of hybrids generated from pummelo and citrus latipes in relation to their attraction to diaphorina citri, the vector of huanglongbing. Metabolites. 2020;10:477. 33255226 10.3390/metabo10120477 PMC7760127

[17] Curtolo M, Pacheco IDS, Boava LP, Takita MA, Granato LM, Galdeano DM, et al. Wide-ranging transcriptomic analysis of Poncirus trifoliata, Citrus sunki, Citrus sinensis and contrasting hybrids reveals HLB tolerance mechanisms. Sci Rep. 2020;10:20865. 33257732 10.1038/s41598-020-77840-2 PMC7705011

[18] Peng Z, Bredeson JV, Wu GA, Shu S, Rawat N, Du D, et al. A chromosome-scale reference genome of trifoliate orange (Poncirus trifoliata) provides insights into disease resistance, cold tolerance and genome evolution in Citrus. Plant J. 2020;104:1215–32. 32985030 10.1111/tpj.14993 PMC7756384

[19] Ramadugu C, Keremane ML, Halbert SE, Duan YP, Roose ML, Stover E, et al. Long-term field evaluation reveals huanglongbing resistance in citrus relatives. Plant Dis. 2016;100:1858–69. 30682983 10.1094/PDIS-03-16-0271-RE

[20] Killiny N, Jones SE, Nehela Y, Hijaz F, Dutt M, Gmitter FG, et al. All roads lead to Rome: towards understanding different avenues of tolerance to huanglongbing in citrus cultivars. Plant Physiol Biochem. 2018;129:1–10. 29783096 10.1016/j.plaphy.2018.05.005

[21] Alves MN, Lopes SA, Raiol-Junior LL, Wulff NA, Girardi EA, Ollitrault P, et al. Resistance to ‘Candidatus Liberibacter Asiaticus,’the huanglongbing associated bacterium, in sexually and/or graft-compatible citrus relatives. Front Plant Sci. 2021;11:617664. 33488659 10.3389/fpls.2020.617664 PMC7820388

[22] Felisberto PADC, Girardi EA, Peña L, Felisberto G, Beattie GA, Lopes SA. Unsuitability of indigenous south American Rutaceae as potential hosts of Diaphorina citri. Pest Manag Sci. 2019;75:1911–20. 30565375 10.1002/ps.5304

[23] Grosser JW, Kainth D, Dutt M. Production of colchicine-induced autotetraploids in pummelo (Citrus grandis Osbeck) through indirect organogenesis. HortScience. 2014;49:944–8.

[24] Kainth D, Grosser JW. Induction of autotetraploids in pummelo (Citrus grandis L. Osbeck) through colchicine treatment of meristematically active seeds in vitro. Proc Fla State Hortic Soc. 2010;123:44–8.

[25] Dutt M, Mahmoud LM, Grosser JW. Field performance of ‘valencia’sweet orange trees grafted onto pummelo interstocks and swingle citrumelo rootstocks under huanglongbing (HLB) endemic conditions. Horticulturae. 2023;9:719.

[26] Gaikwad PN, Singh J, Sidhu GS. Identification and diversity analysis of interspecific citrus rootstock hybrids with combination of morphological traits and microsatellite markers. Hortic Environ Biotechnol. 2024;65:539–65.

[27] Singh J, Sharma A, Sharma V, Gaikwad PN, Sidhu GS, Kaur G, et al. Comprehensive genome-wide identification and transferability of chromosome-specific highly variable microsatellite markers from citrus species. Sci Rep. 2023;13:10919. 37407627 10.1038/s41598-023-37024-0 PMC10322976

[28] Wang Z, Yin Y, Hu H, Yuan Q, Peng G, Xia Y. Development and application of molecular-based diagnosis for ‘Candidatus Liberibacter Asiaticus’, the causal pathogen of citrus huanglongbing. Plant Pathol. 2006;55:630–8.

[29] Lichtenthaler HK, Wellburn AR. Determinations of total carotenoids and chlorophylls a and b of leaf extracts in different solvents. Biochem Soc Trans. 1983;603:591–2.

[30] Singleton VL, Rossi JA. Colorimetry of total phenolics with phosphomolybdic-phosphotungstic acid reagents. Am J Enol Vitic. 1965;16:144–58.

[31] Aktumsek A, Zengin G, Guler GO, Cakmak YS, Duran A. Antioxidant potentials and anticholinesterase activities of methanolic and aqueous extracts of three endemic Centaurea L. species. Food Chem Toxicol. 2013;55:290–6. 23357566 10.1016/j.fct.2013.01.018

[32] Chen Y, Chen Y, Shi C, Huang Z, Zhang Y, Li S, et al. SOAPnuke: a MapReduce acceleration-supported software for integrated quality control and preprocessing of high-throughput sequencing data. Gigascience. 2018;7:1–6. 29220494 10.1093/gigascience/gix120 PMC5788068

[33] Love MI, Huber W, Anders S. Moderated estimation of Fold change and dispersion for RNA-seq data with DESeq2. Genome Biol. 2014;15:550. 25516281 10.1186/s13059-014-0550-8 PMC4302049

[34] Supek F, Bosnjak M, Skunca N, Smuc T. REVIGO summarizes and visualizes long lists of gene ontology terms. PLoS ONE. 2011;6:e21800. 21789182 10.1371/journal.pone.0021800 PMC3138752

[35] Thimm O, Blasing O, Gibon Y, Nagel A, Meyer S, Kruger P, et al. MAPMAN: a user-driven tool to display genomics data sets onto diagrams of metabolic pathways and other biological processes. Plant J. 2004;37:914–39. 14996223 10.1111/j.1365-313x.2004.02016.x

[36] Usadel B, Nagel A, Steinhauser D, Gibon Y, Blasing OE, Redestig H, et al. PageMan: an interactive ontology tool to generate, display, and annotate overview graphs for profiling experiments. BMC Bioinform. 2006;7:535. 10.1186/1471-2105-7-535 PMC1766370 17176458

[37] Livak KJ, Schmittgen TD. Analysis of relative gene expression data using real-time quantitative PCR and the 2(-Delta Delta C(T)) method. Methods. 2001;25:402–8. 11846609 10.1006/meth.2001.1262

[38] Ward JH. Hierarchical grouping to optimize an objective function. J Am Stat Assoc. 1963;58:236–44.

[39] Rawat N, Kiran SP, Du D, Gmitter FG, Deng Z. Comprehensive meta-analysis, co-expression, and miRNA nested network analysis identifies gene candidates in citrus against Huanglongbing disease. BMC Plant Biol. 2015;15:184. 26215595 10.1186/s12870-015-0568-4 PMC4517500

[40] Arce-Leal AP, Bautista R, Rodriguez-Negrete EA, Manzanilla-Ramirez MA, Velazquez-Monreal JJ, Santos-Cervantes ME, et al. Gene expression profile of Mexican lime (Citrus aurantifolia) trees in response to huanglongbing disease caused by candidatus Liberibacter Asiaticus. Microorganisms. 2020;8:528. 32272632 10.3390/microorganisms8040528 PMC7232340

[41] Liu C, Chang X, Li F, Yan Y, Zuo X, Huang G, et al. Transcriptome analysis of Citrus sinensis reveals potential responsive events triggered by Candidatus Liberibacter asiaticus. Protoplasma. 2024;261:499–512. 38092896 10.1007/s00709-023-01911-0

[42] Li R, Wang X, Hu Y, Huang G. Analysis of huanglongbing-associated RNA-seq data reveals disturbances in biological processes within Citrus spp. triggered by Candidatus Liberibacter asiaticus infection. Front Plant Sci. 2024;15:1388163. 38660443 10.3389/fpls.2024.1388163 PMC11039969

[43] Singh A, Panwar R, Mittal P, Hassan MI, Singh IK. Plant cytochrome P450s: role in stress tolerance and potential applications for human welfare. Int J Biol Macromol. 2021;184:874–86. 34175340 10.1016/j.ijbiomac.2021.06.125

[44] Ro DK, Arimura GI, Lau SY, Piers E, Bohlmann J. Loblolly pine abietadienol/abietadienal oxidase PtAO (CYP720B1) is a multifunctional, multisubstrate cytochrome P450 monooxygenase. Proc Natl Acad Sci. 2005;102:8060–5. 15911762 10.1073/pnas.0500825102 PMC1138258

[45] Wu G, Wang W. Recent advances in understanding the role of two mitogen-activated protein kinase cascades in plant immunity. J Exp Bot. 2024;75:2256–65. 38241698 10.1093/jxb/erae020

[46] Divekar PA, Rani V, Majumder S, Karkute SG, Molla KA, Pandey KK, et al. Protease inhibitors: an induced plant defense mechanism against herbivores. J. Plant Growth Regul. 2023;42(10):6057–73

[47] Yu X, Killiny N. The secreted salivary proteome of Asian citrus psyllid Diaphorina citri. Physiol Entomol. 2018;43:324–33.

[48] Suman S, Bagal D, Jain D, Singh R, Singh IK, Singh A. Biotic stresses on plants: reactive oxygen species generation and antioxidant mechanism. In: Aftab T and Hakeem KR, editors. Frontiers in plant-soil interaction. Molecular Insights into Plant Adaptation. Elsevier; 2021. p.381–411.

[49] Pandey VP, Awasthi M, Singh S, Tiwari S, Dwivedi UN. A comprehensive review on function and application of plant peroxidases. Biochem Anal Biochem. 2017;6:308.

[50] Govindan G, Sandhiya K, Alphonse V, Somasundram S. Role of Germin-like proteins (GLPs) in biotic and abiotic stress responses in major crops: a review on plant defense mechanisms and stress tolerance. Plant Mol Biol Rep. 2024;42:450–68.

[51] Wang Y, Zhou L, Yu X, Stover E, Luo F, Duan Y. Transcriptome profiling of huanglongbing (HLB) tolerant and susceptible citrus plants reveals the role of basal resistance in HLB tolerance. Front Plant Sci. 2016;7:933. 27446161 10.3389/fpls.2016.00933 PMC4923198

[52] Hu Y, Zhong X, Liu X, Lou B, Zhou C, Wang X. Comparative transcriptome analysis unveils the tolerance mechanisms of Citrus hystrix in response to ‘Candidatus Liberibacter asiaticus’ infection. PLoS ONE. 2017;12:e0189229. 29232716 10.1371/journal.pone.0189229 PMC5726760

[53] Fan J, Chen C, Yu Q, Khalaf A, Achor DS, Brlansky RH, et al. Comparative transcriptional and anatomical analyses of tolerant rough lemon and susceptible sweet orange in response to ‘Candidatus Liberibacter asiaticus’ infection. Mol Plant-Microbe Interact. 2012;25:1396–407. 22809274 10.1094/MPMI-06-12-0150-R

[54] Rashid A. Defense responses of plant cell wall non-catalytic proteins against pathogens. Physiol Mol Plant Pathol. 2016;94:38–46.

[55] Achor D, Etxeberria E, Wang N, Folimonova S, Chung K, Albrigo L. Sequence of anatomical symptom observations in citrus affected with huanglongbing disease. Plant Pathol J. 2010;9:56–64.

[56] Mattos-Jr D, Kadyampakeni DM, da Silva JR, Vashisth T, Boaretto RM. Reciprocal effects of huanglongbing infection and nutritional status of citrus trees: a review. Trop Plant Pathol. 2020;45:586–96.

[57] Xu M, Li Y, Zheng Z, Dai Z, Tao Y, Deng X. Transcriptional analyses of mandarins seriously infected by ‘Candidatus Liberibacter asiaticus’. PLoS ONE. 2015;10:e0133652. 26196297 10.1371/journal.pone.0133652 PMC4511008

[58] Ferrer JL, Austin MB, Stewart C, Noel JP. Structure and function of enzymes involved in the biosynthesis of phenylpropanoids. Plant Physiol Biochem. 2008;46:356–70. 18272377 10.1016/j.plaphy.2007.12.009 PMC2860624

[59] Nehela Y, Hijaz F, Elzaawely AA, El-Zahaby HM, Killiny N. Citrus phytohormonal response to Candidatus Liberibacter asiaticus and its vector Diaphorina citri. Physiol Mol Plant Pathol. 2018;102:24–35.

[60] Eulgem T, Somssich IE. Networks of WRKY transcription factors in defense signaling. Curr Opin Plant Biol. 2007;10:366–71. 17644023 10.1016/j.pbi.2007.04.020

[61] Kim JS, Sagaram US, Burns JK, Li JL, Wang N. Response of sweet orange (Citrus sinensis) to ‘Candidatus Liberibacter asiaticus’ infection: microscopy and microarray analyses. Phytopathology. 2009;99:50–7. 19055434 10.1094/PHYTO-99-1-0050

[62] Baena-Gonzalez E, Rolland F, Thevelein JM, Sheen J. A central integrator of transcription networks in plant stress and energy signalling. Nature. 2007;448:938–42. 17671505 10.1038/nature06069

