# Supplementary material for: Transcriptomic and biochemical analysis of pummelo x finger lime hybrids in response to Huanglongbing (HLB)
Source: BMC Plant Biol. 2025 Feb 20;25:235. doi: 10.1186/s12870-025-06211-8 (PMC11841000; doi:10.1186/s12870-025-06211-8)
Supplement: Supplementary file 1 — Supplementary Material 1 [file 12870_2025_6211_MOESM1_ESM.docx]

**Transcriptomic and Biochemical Analysis of Pummelo-Finger Lime Hybrids in Response to Huanglongbing (HLB) disease**

Lamiaa M. Mahmoud^1^, Jaideep Deol^1^, Jude W. Grosser^1,2^, Nabil Killiny^3^, Manjul Dutt^1,2^*

^1^ Department of Horticultural Sciences, Citrus Research and Education Center, University of Florida, Lake Alfred, FL, USA.

^2^ Plant Breeding Graduate Program, University of Florida, Gainesville, Florida, USA.

^3^ Department of Plant Pathology, Citrus Research and Education Center, University of Florida, Lake Alfred, FL, USA.

* [manjul@ufl.edu](mailto:manjul@ufl.edu)

**
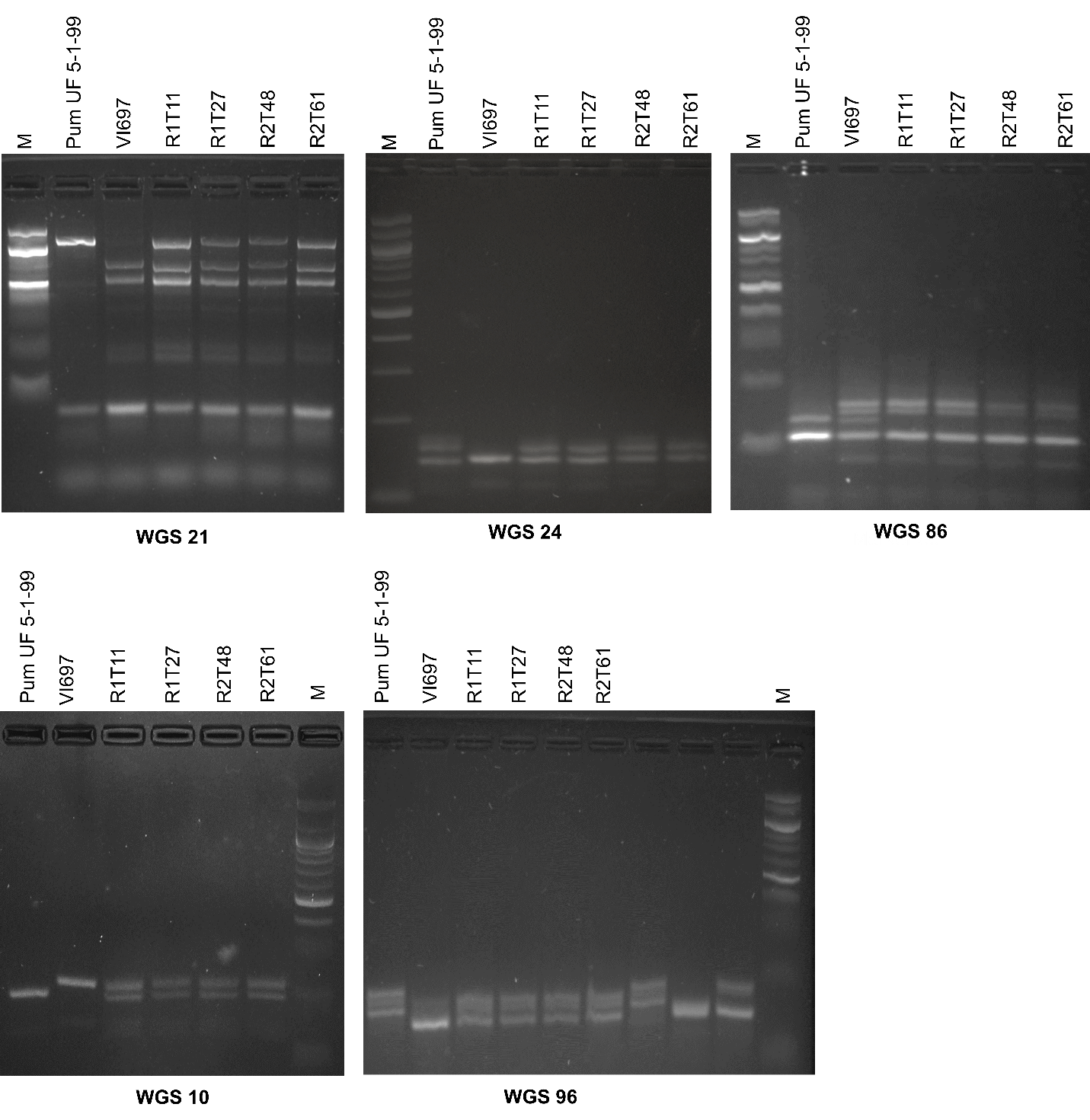
**

**Figure S1:** Hybrid confirmation on for Pummelo-Finger Lime Hybrids using simple sequence repeat (SSR) markers.

**Table S1.** List of the primer sequences used for Simple Sequence Repeats (SSR) Molecular Marker Analysis.

| **Primer name** | **Forward sequence** | **Reverse sequence** | **Annealing temp. (C°)** |
| --- | --- | --- | --- |
| WGS- 10 | CAGCCTAAGGTACGAGGAATAC | TGATGATACAGTGGCGATTACA | 52 |
| WGS- 21 | AAATTACGGAGCTCCTCACG | ATGAGCTCTTGCCATTACACT | 52 |
| WGS- 24 | GCATCCTTCTCAGTATGACGTG | TCCTACTCTCTATCCCTCTGTCTCA | 54 |
| WGS- 86 | TGTCGATTTCGGTTCTTCTTCT | GATGGTAAGATTGGTGATGATGATG | 52 |
| WGS- 96 | TACCCTGCTGCTGTTTTGC | TTCTCAACTGGGAAGCTCCT | 54 |

**Table S2.** List of the primer sequences used for SYBR Green-based qPCR assay.

| Gene ID | Sequence | |
| --- | --- | --- |
| Ciclev10028904m | F | GTT CGT GCT CGT GAG AGA AT |
|  | R | CAT CTC CGA TGA CGA GAT TGA C |
| Ciclev10006920m | F | GTT CTA ACA TAC GGA GGG AAG TC |
|  | R | AAG GCT CGT GTT ACT CAT AGT TT |
| Ciclev10003684m | F | CCA TCA TCC AGT GTT CCA AAT C |
|  | R | CAG CTC CAG AAG ACA CTA CAA |
| Ciclev10007746m | F | GTT CCC ACC ATG GCC TAT TT |
|  | R | CAA CTC TTG TCG CTC CTT CTC |
| Ciclev10005766m | F | ATC TTG GTG ATT GAG GCA TAC A |
|  | R | AAG GCA AAG TAC CCA CTA AGG |
| Ciclev10022911m | F | GAA ACC CTG GAG CTC AAA CT |
|  | R | CGG AAG GAC AGT GAA CAA TCT |
| Ciclev10032840m | F | GAA CTT GCC GAT GAG CTT AGA |
|  | R | TAT GCG TCG AAA TCA CCT TCA |
| Ciclev10023557m | F | GAA GCA TCC CGA GAC TAC TAT TT |
|  | R | ACA TCA AGT GGG CAG AGT TC |

**Table S3.** Comparative Gene Ontology (GO) classifications of commonly expressed functionally annotated DEGs from two cross combinations in the Finger lime hybrids transcriptome. The genes corresponded to three main categories, cellular component, molecular function, and biological process.

| **GO term ID*** | **GO term** | **Subgroup** | **Gene number** |
| --- | --- | --- | --- |
| GO:0005634 | Nucleus | Cellular component | 113 |
| GO:0005886 | Plasma membrane |  | 87 |
| GO:0005737 | Cytoplasm |  | 60 |
| GO:0005829 | Cytosol |  | 48 |
| GO:0009507 | Chloroplast |  | 44 |
| GO:0005576 | Extracellular region |  | 33 |
| GO:0005618 | Cell wall |  | 26 |
| GO:0005739 | Mitochondrion |  | 26 |
| GO:0048046 | Apoplast |  | 23 |
| GO:0009506 | Plasmodesma |  | 21 |
| GO:0005783 | Endoplasmic reticulum |  | 20 |
| GO:0005789 | Endoplasmic reticulum membrane |  | 19 |
| GO:0005794 | Golgi apparatus |  | 19 |
| GO:0009570 | Chloroplast stroma |  | 18 |
| GO:0000139 | Golgi membrane |  | 17 |
| GO:0005773 | Vacuole |  | 15 |
| GO:0016020 | Membrane |  | 15 |
| GO:0009536 | Plastid |  | 14 |
| GO:0046872 | Metal ion binding | Molecular function | 72 |
| GO:0005524 | ATP binding |  | 63 |
| GO:0003700 | DNA-binding transcription factor activity |  | 39 |
| GO:0003677 | DNA binding |  | 31 |
| GO:0005506 | Iron ion binding |  | 24 |
| GO:0020037 | Heme binding |  | 24 |
| GO:0000976 | Transcription cis-regulatory region binding |  | 15 |
| GO:0004674 | Protein serine/threonine kinase activity |  | 15 |
| GO:0008270 | Zinc ion binding |  | 15 |
| GO:0016491 | Oxidoreductase activity |  | 12 |
| GO:0003729 | mRNA binding |  | 12 |
| GO:0016705 | Oxidoreductase activity |  | 10 |
| GO:0004497 | Monooxygenase activity |  | 10 |
| GO:0043565 | Sequence-specific DNA binding |  | 10 |
| GO:0030246 | Carbohydrate binding |  | 9 |
| GO:0043621 | Protein self-association |  | 8 |
| GO:0016757 | Glycosyltransferase activity |  | 8 |
| GO:0005525 | GTP binding |  | 8 |
| GO:0006355 | Regulation of DNA-templated transcription | Biological process | 28 |
| GO:0071555 | Cell wall organization |  | 21 |
| GO:0006952 | Defense response |  | 20 |
| GO:0009651 | Response to salt stress |  | 18 |
| GO:0009737 | Response to abscisic acid |  | 18 |
| GO:0005975 | Carbohydrate metabolic process |  | 16 |
| GO:0009409 | Response to cold |  | 13 |
| GO:0009414 | Response to water deprivation |  | 12 |
| GO:0042742 | Defense response to bacterium |  | 12 |
| GO:0016567 | Protein ubiquitination |  | 11 |
| GO:0045490 | Pectin catabolic process |  | 9 |
| GO:0046777 | Protein autophosphorylation |  | 9 |
| GO:0050832 | Defense response to fungus |  | 9 |
| GO:0009834 | Plant-type secondary cell wall biogenesis |  | 8 |
| GO:0009408 | Response to heat |  | 8 |
| GO:0045892 | Negative regulation of DNA-templated transcription |  | 8 |
| GO:0009611 | Response to wounding |  | 8 |
| GO:0009738 | Abscisic acid-activated Signaling pathway |  | 8 |

**p* value < 0.05 for all the studied GO terms.

**Table S4.**

| **Gene symbol** | **Log2fc** | ***p* value** | Function/Annotation |
| --- | --- | --- | --- |
| Ciclev10010007m | 9 | 0.0039 | No specific annotation |
| Ciclev10022911m | 5 | 0.0004 | Kunitz Family Trypsin and Protease Inhibitor Protein-Related |
| Ciclev10026966m | 5 | 0.0247 | Abieta-7,13-dien-18-ol Hydroxylase / CYP720B1 |
| Ciclev10022118m | 5 | 0.0045 | Germin-Like Protein Subfamily 1 Member 13-Related |
| Ciclev10022211m | 5 | 0.0225 | Trypsin and Protease Inhibitor (Kunitz_legume) |
| Ciclev10022906m | 4 | 0.0167 | Potato Inhibitor I Family (potato_inhibit) |
| Ciclev10027501m | 4 | 0.0404 | Oxidoreductase, 2OG-FE II Oxygenase Family Protein |
| Ciclev10005432m | 4 | 0.0378 | Peroxidase / Lactoperoxidase |
| Ciclev10023996m | 3 | 0.0053 | Oxidoreductase, 2OG-FE II Oxygenase Family Protein |
| Ciclev10026390m | 3 | 0.0277 | 5 kDa Heat Shock Protein, Mitochondrial |
| Ciclev10031245m | 3 | 0.0001 | Flavonoid 3'-Monooxygenase / Flavonoid 3'-Hydroxylase |
| Ciclev10028831m | 3 | 0.0020 | Basic Endochitinase B |
| Ciclev10006042m | 3 | 0.0203 | Protein Brother of FT and TFL 1 |
| Ciclev10016018m | 3 | 0.0421 | F2401_16 |
| Ciclev10001560m | 3 | 0.0494 | No specific annotation |
| Ciclev10011227m | 3 | 0.0049 | ATP-DEPENDENT ZINC METALLOPROTEASE FTSH 6, CHLOROPLASTIC |
| Ciclev10026748m | 3 | 0.0450 | No function available |
| Ciclev10028461m | 3 | 0.0001 | No function available |
| Ciclev10027059m | 3 | 0.0005 | Flavin-binding monooxygenase-like (FMO-like) |
| Ciclev10028964m | 3 | 0.0147 | BASIC ENDOCHITINASE B |
| Ciclev10005095m | 3 | 0.0001 | WRKY TRANSCRIPTION FACTOR 27-RELATED |
| Ciclev10027953m | 3 | 0.0001 | ATP-BINDING CASSETTE TRANSPORTER |
| Ciclev10031562m | 3 | 0.0101 | Predicted transporter ADD1 (major facilitator superfamily) |
| Ciclev10022111m | 3 | 0.0070 | GERMIN-LIKE PROTEIN SUBFAMILY 1 MEMBER 10-RELATED |
| Ciclev10028272m | 3 | 0.0317 | Lysosomal Pro-Xaa carboxypeptidase / Prolyl carboxypeptidase |
| Ciclev10013206m | 3 | 0.0015 | Angiotensin-converting enzyme 2 / Angiotensin converting enzyme-2 |
| Ciclev10004586m | 3 | 0.0071 | GRAS domain family (GRAS) |
| Ciclev10014810m | 3 | 0.0041 | Tetrahydroberberine oxidase / THB oxidase |
| Ciclev10007040m | 3 | 0.0468 | 3-phytase / Phytate 6-phosphatase |
| Ciclev10003082m | 3 | 0.0128 | No function available |
| Ciclev10020833m | 2 | 0.0049 | NADH-UBIQUINONE OXIDOREDUCTASE FLAVOPROTEIN 1 NDUFV1 |
| Ciclev10027215m | 2 | 0.0136 | No apical meristem-associated C-terminal domain (NAM-associated) |
| Ciclev10004491m | 2 | 0.0013 | PLASTID-LIPID-ASSOCIATED PROTEIN 14, CHLOROPLASTIC-RELATED |
| Ciclev10016109m | 2 | 0.0280 | PDDEXK-like family of unknown function (PDDEXK_6) |
| Ciclev10015568m | 2 | 0.0393 | RELATED TO MULTIFUNCTIONAL CYCLIN-DEPENDENT KINASE-RELATED |
| Ciclev10008450m | 2 | 0.0027 | MONOOXYGENASE |
| Ciclev10002204m | 2 | 0.0038 | F-BOX PROTEIN PP2-B13-RELATED |
| Ciclev10031237m | 2 | 0.0003 | CYTOCHROME P450 78A5-RELATED |
| Ciclev10010537m | 2 | 0.0487 | MONOOXYGENASE |
| Ciclev10002395m | 2 | 0.0152 | Trypsin and protease inhibitor (Kunitz_legume) |
| Ciclev10023446m | 2 | 0.0062 | F21J9 4 |
| Ciclev10000751m | 2 | 0.0141 | MATE EFFLUX FAMILY PROTEIN |
| Ciclev10002994m | 2 | 0.0460 | No function available |
| Ciclev10012894m | 2 | 0.0268 | No function available |
| Ciclev10004583m | 2 | 0.0002 | No function available |
| Ciclev10007191m | 2 | 0.0010 | MATE EFFLUX FAMILY PROTEIN |
| Ciclev10024414m | 2 | 0.0461 | No function available |
| Ciclev10019727m | 2 | 0.0002 | Cytochrome P450 CYP2 subfamily |
| Ciclev10031637m | 2 | 0.0023 | No specific annotation provided |
| Ciclev10011671m | 2 | 0.0003 | PF03140 - Plant protein of unknown function (DUF247) |
| Ciclev10023612m | 2 | 0.0039 | SF45 - CALCINEURIN B-LIKE PROTEIN 4 |
| Ciclev10000844m | 2 | 0.0062 | KOG0156 - Cytochrome P450 CYP2 subfamily |
| Ciclev10020818m | 2 | 0.0152 | SF103 - O-METHYLTRANSFERASE |
| Ciclev10014204m | 2 | 0.0213 | 1 13 11 12 - Linoleate 13S-lipoxygenase / Lipoxidase |
| Ciclev10006881m | 2 | 0.0121 | No specific annotation provided |
| Ciclev10004039m | 2 | 0.0084 | PF00069//PF08263//PF13855 - Protein kinase domain // Leucine rich repeat N-terminal domain (LRRNT_2) // Leucine rich repeat (LRR_8) |
| Ciclev10008091m | 2 | 0.0003 | 1 2 1 9 - Glyceraldehyde-3-phosphate dehydrogenase (NADP(+)) / Triosephosphate dehydrogenase |
| Ciclev10005157m | 2 | 0.0099 | 1 14 11 12 - Gibberellin-44 dioxygenase / Gibberellin A44 oxidase |
| Ciclev10019053m | 2 | 0.0259 | SF41 - FERRIC REDUCTION OXIDASE 2-RELATED |
| Ciclev10033223m | 2 | 0.0061 | No specific annotation provided |
| Ciclev10000806m | 2 | 0.0333 | SF50 - SYNAPTOTAGMIN-1-RELATED |
| Ciclev10010560m | 2 | 0.0192 | No specific annotation provided |
| Ciclev10003965m | 2 | 0.0167 | PF00069//PF00560//PF13855 - Protein kinase domain // Leucine Rich Repeat (LRR_1) // Leucine rich repeat (LRR_8) |
| Ciclev10024587m | 2 | 0.0430 | SF86 - CYTOCHROME P450 71B21-RELATED |
| Ciclev10030579m | 2 | 0.0241 | Xenobiotic-transporting ATPase / Steroid-transporting ATPase |
| Ciclev10020552m | 2 | 0.0269 | PF00560//PF13855 - Leucine Rich Repeat (LRR_1) // Leucine rich repeat (LRR_8) |
| Ciclev10007772m | 2 | 0.0043 | Cycloartenol synthase / 2,3-epoxysqualene--cycloartenol cyclase |
| Ciclev10025382m | 2 | 0.0033 | KOG0156 - Cytochrome P450 CYP2 subfamily |
| Ciclev10033071m | 2 | 0.0007 | SF278 - THIOREDOXIN H7-RELATED |
| Ciclev10000808m | 2 | 0.0086 | 1 3 3 8 - Tetrahydroberberine oxidase / THB oxidase |
| Ciclev10016616m | 2 | 0.0028 | No specific annotation provided |
| Ciclev10022480m | 2 | 0.0449 | SF59 - ACHAETE-SCUTE TRANSCRIPTION FACTOR-RELATED |
| Ciclev10025045m | 2 | 0.0095 | SF48 - ABC TRANSPORTER F FAMILY MEMBER 2-RELATED |
| Ciclev10010416m | 2 | 0.0019 | 5 4 99 8 - Cycloartenol synthase / 2,3-epoxysqualene--cycloartenol cyclase |
| Ciclev10021866m | 2 | 0.0042 | BRASSINOSTEROID METABOLIC PATHWAY PROTEIN BEN1 |
| Ciclev10021154m | 2 | 0.0035 | SF169 - FRUCTOKINASE-5-RELATED |
| Ciclev10008749m | 2 | 0.0013 | NUCLEOTIDE-DIPHOSPHO-SUGAR TRANSFERASE DOMAIN-CONTAINING PROTEIN-RELATED |
| Ciclev10003524m | 2 | 0.0425 | No specific annotation provided |
| Ciclev10022829m | 2 | 0.0013 | No specific annotation provided |
| Ciclev10031307m | 2 | 0.0481 | 1 14 13 124 - Phenylalanine N-monooxygenase / Phenylalanine N-hydroxylase |
| Ciclev10023513m | 2 | 0.0424 | PF00560//PF13855 - Leucine Rich Repeat (LRR_1) // Leucine rich repeat (LRR_8) |
| Ciclev10030176m | 2 | 0.0370 | PF00560//PF08263//PF13855 - Leucine Rich Repeat (LRR_1) // Leucine rich repeat N-terminal domain (LRRNT_2) // Leucine rich repeat (LRR_8) |
| Ciclev10009259m | 2 | 0.0001 | Lysophospholipase / Phospholipase B |
| Ciclev10015385m | 2 | 0.0002 | SF237 - DNAJ HOMOLOG SUBFAMILY C MEMBER |
| Ciclev10025932m | 2 | 0.0001 | F18B13.21 PROTEIN-RELATED |
| Ciclev10020323m | 2 | 0.0005 | SF165 - TRANSCRIPTION FACTOR PIF7 |
| Ciclev10016803m | 2 | 0.0397 | MYB-LIKE DNA-BINDING PROTEIN MYB |
| Ciclev10016729m | 2 | 0.0145 | Predicted protein tyrosine phosphatase |
| Ciclev10019723m | 2 | 0.0048 | WRC (WRC) // QLQ (QLQ) |
| Ciclev10004779m | 2 | 0.0442 | SF30 - SUGAR TRANSPORT PROTEIN 3 |
| Ciclev10009331m | 2 | 0.0002 | SF6 - EXPRESSED PROTEIN |
| Ciclev10022303m | 2 | 0.0066 | SF558 - MYB-LIKE DNA-BINDING PROTEIN MYB |
| Ciclev10009522m | 2 | 0.0216 | SF257 - HOMEOBOX-LEUCINE ZIPPER PROTEIN ATHB-52 |
| Ciclev10029108m | 2 | 0.0042 | TRANSCRIPTION FACTOR MYB48-RELATED |
| Ciclev10001348m | 2 | 0.0015 | SF118 - ALPHA-AMYLASE 2-RELATED |
| Ciclev10033608m | 2 | 0.0362 | PF00560//PF07714//PF13855 - Leucine Rich Repeat (LRR_1) // Protein tyrosine kinase (Pkinase_Tyr) // Leucine rich repeat (LRR_8) |
| Ciclev10026133m | 2 | 0.0109 | SF3 - GB |
| Ciclev10000884m | 2 | 0.0098 | SF57 - ENT-KAURENE OXIDASE, CHLOROPLASTIC |
| Ciclev10031002m | 2 | 0.0095 | SF26 - SISTER CHROMATID COHESION 1 PROTEIN 1 |
| Ciclev10024390m | 2 | 0.0246 | Cytochrome P450 CYP2 subfamily |
| Ciclev10021505m | 2 | 0.0007 | TETRAPYRROLE-BINDING PROTEIN, CHLOROPLAST |
| Ciclev10029369m | 2 | 0.0110 | ADP RIBOSYLATION FACTOR-RELATED |
| Ciclev10011554m | 2 | 0.0298 | PROTEIN REVEILLE 1-RELATED |
| Ciclev10013176m | 2 | 0.0209 | Potato inhibitor I family (potato_inhibit) |
| Ciclev10023611m | 2 | 0.0036 | CHITINASE-RELATED |
| Ciclev10009511m | 1 | 0.0302 | No specific annotation provided |
| Ciclev10013069m | 1 | 0.0016 | No specific annotation provided |
| Ciclev10030339m | 1 | 0.0257 | ALDO-KETO REDUCTASE 1-RELATED |
| Ciclev10001179m | 1 | 0.0091 | ENT-KAURENE OXIDASE, CHLOROPLAST |
| Ciclev10002968m | 1 | 0.0013 | No specific annotation provided |
| Ciclev10006074m | 1 | 0.0236 | Pathogenesis-related protein Bet v I family |
| Ciclev10008047m | 1 | 0.0012 | Premnaspirodiene oxygenase / Hyoscymus muticus premnaspirodiene oxygenase |
| Ciclev10011658m | 1 | 0.0001 | RHOMBOID FAMILY PROTEIN |
| Ciclev10023966m | 1 | 0.0376 | CYTOCHROME P450 71B21-RELATED |
| Ciclev10025175m | 1 | 0.0480 | Leucine Rich Repeat / Protein tyrosine kinase / Leucine rich repeat |
| Ciclev10013710m | 1 | 0.0018 | HYDROPHOBIC PROTEIN RCI2A |
| Ciclev10014538m | 1 | 0.0133 | SULFATE TRANSPORTER 3-RELATED |
| Ciclev10031759m | 1 | 0.0033 | METAL TOLERANCE PROTEIN 11 |
| Ciclev10021170m | 1 | 0.0011 | MITOGEN-ACTIVATED PROTEIN KINASE KINASE 7-RELATED |
| Ciclev10033181m | 1 | 0.0095 | Angiotensin-converting enzyme 2 / Angiotensin converting enzyme-2 |
| Ciclev10009866m | 1 | 0.0002 | Cytochrome P450 CYP2 subfamily |
| Ciclev10009083m | 1 | 0.0297 | SPX DOMAIN-CONTAINING PROTEIN 1-RELATED |
| Ciclev10006298m | 1 | 0.0009 | No specific annotation provided |
| Ciclev10000809m | 1 | 0.0018 | Cytochrome P450 CYP2 subfamily |
| Ciclev10001522m | 1 | 0.0485 | Protein tyrosine kinase / Domain of unknown function |
| Ciclev10017717m | 1 | 0.0036 | Predicted hydrolase (HIT family) |
| Ciclev10032778m | 1 | 0.0025 | ALPHA/BETA HYDROLASE RELATED PROTEIN |
| Ciclev10000613m | 1 | 0.0331 | Protein of unknown function (DUF668) |
| Ciclev10003538m | 1 | 0.0301 | Protein kinase domain / Leucine Rich Repeat |
| Ciclev10025330m | 1 | 0.0027 | Plant protein of unknown function (DUF247) |
| Ciclev10004104m | 1 | 0.0001 | CYTOCHROME P450 71B21-RELATED |
| Ciclev10017588m | 1 | 0.0016 | hAT family C-terminal dimerisation region / Domain of unknown function |
| Ciclev10021622m | 1 | 0.0480 | AP2 domain |
| Ciclev10023227m | 1 | 0.0121 | No specific annotation provided |
| Ciclev10014162m | 1 | 0.0105 | No specific annotation provided |
| Ciclev10006216m | 1 | 0.0280 | PROTEIN PROTON GRADIENT REGULATION 5, CHLOROPLAST |
| Ciclev10020658m | 1 | 0.0002 | No specific annotation provided |
| Ciclev10013742m | 1 | 0.0096 | No specific annotation provided |
| Ciclev10002706m | 1 | 0.0034 | EF-HAND CALCIUM-BINDING DOMAIN CONTAINING PROTEIN |
| Ciclev10000902m | 1 | 0.0043 | ENT-KAURENE OXIDASE, CHLOROPLAST |
| Ciclev10026549m | 1 | 0.0016 | HISTONE H1 |
| Ciclev10007057m | 1 | 0.0143 | BIDIRECTIONAL SUGAR TRANSPORTER SWEET3 |
| Ciclev10030965m | 1 | 0.0014 | Protein kinase domain / Salt stress response/antifungal |
| Ciclev10011386m | 1 | 0.0117 | WRKY TRANSCRIPTION FACTOR 1-RELATED |
| Ciclev10014790m | 1 | 0.0204 | Alpha-farnesene synthase / AFS1 |
| Ciclev10002231m | 1 | 0.0015 | F14N23 10 |
| Ciclev10032884m | 1 | 0.0442 | No specific annotation provided |
| Ciclev10015503m | 1 | 0.0075 | PROTOCHLOROPHYLLIDE-DEPENDENT TRANSLOCON COMPONENT 52, CHLOROPLASTIC |
| Ciclev10019997m | 1 | 0.0139 | PYRUVATE KINASE |
| Ciclev10002326m | 1 | 0.0181 | Function not specified |
| Ciclev10024674m | 1 | 0.0061 | Germacradienol synthase / Germacradienol/germacrene-D synthase |
| Ciclev10007334m | 1 | 0.0323 | Leucine Rich Repeat (LRR) domain-containing protein |
| Ciclev10012836m | 1 | 0.0004 | GRAS domain family (GRAS) |
| Ciclev10006265m | 1 | 0.0003 | Function not specified |
| Ciclev10009622m | 1 | 0.0043 | APOLIPOPROTEIN D |
| Ciclev10002381m | 1 | 0.0147 | Function not specified |
| Ciclev10031674m | 1 | 0.0033 | [Protein-PII] uridylyltransferase / Uridylyl removing enzyme |
| Ciclev10015513m | 1 | 0.0009 | PURINE PERMEASE 10-RELATED |
| Ciclev10022962m | 1 | 0.0179 | Putative AtpZ or ATP-synthase-associated (ATP-synt_Z) |
| Ciclev10028879m | 1 | 0.0057 | Fantastic Four meristem regulator (FAF) |
| Ciclev10008073m | 1 | 0.0004 | Cytochrome P450 CYP2 subfamily |
| Ciclev10029754m | 1 | 0.0002 | EXPRESSED PROTEIN |
| Ciclev10009213m | 1 | 0.0491 | B-BOX DOMAIN PROTEIN 26-RELATED |
| Ciclev10006888m | 1 | 0.0470 | C3HC4 ZINC FINGER DOMAIN-CONTAINING PROTEIN-RELATED |
| Ciclev10028701m | 1 | 0.0231 | Function not specified |
| Ciclev10031389m | 1 | 0.0003 | GRAS domain family (GRAS) |
| Ciclev10001604m | 1 | 0.0057 | Function not specified |
| Ciclev10005127m | 1 | 0.0019 | ASPARTYL PROTEASE-LIKE PROTEIN |
| Ciclev10026060m | 1 | 0.0348 | Ribonucleoside-diphosphate reductase / Ribonucleotide reductase |
| Ciclev10014601m | 1 | 0.0461 | MAC/Perforin domain (MACPF) |
| Ciclev10001030m | 1 | 0.0269 | 3-methyl-2-oxobutanoate dehydrogenase (2-methylpropanoyl-transferring) / Dehydrogenase, branched chain alpha-keto acid |
| Ciclev10005233m | 1 | 0.0004 | Helix-loop-helix DNA-binding domain (HLH) |
| Ciclev10014857m | 1 | 0.0020 | Tetrahydroberberine oxidase / THB oxidase |
| Ciclev10005067m | 1 | 0.0101 | Function not specified |
| Ciclev10006093m | 1 | 0.0001 | Function not specified |
| Ciclev10000623m | 1 | 0.0352 | Salt stress response/antifungal (Stress-antifung), Protein tyrosine kinase (Pkinase_Tyr), Domain of unknown function (DUF3403) |
| Ciclev10001096m | 1 | 0.0009 | PHOSPHATIDYLINOSITOL-4-PHOSPHATE 5-KINASE RELATED |
| Ciclev10027720m | 1 | 0.0210 | DOUBLE CLP-N MOTIF-CONTAINING P-LOOP NUCLEOSIDE TRIPHOSPHATE HYDROLASE DOMAIN-CONTAINING PROTEIN-RELATED |
| Ciclev10005574m | 1 | 0.0021 | NADH-UBIQUINONE OXIDOREDUCTASE FLAVOPROTEIN 1 (NDUFV1) |
| Ciclev10031846m | 1 | 0.0013 | AP2/ERF AND B3 DOMAIN-CONTAINING TRANSCRIPTION REPRESSOR RAV2-RELATED |
| Ciclev10019581m | 1 | 0.0005 | OXIDOREDUCTASE, 2OG-FE(II) OXYGENASE FAMILY PROTEIN |
| Ciclev10005151m | 1 | 0.0151 | TREHALOSE-PHOSPHATE PHOSPHATASE F-RELATED |
| Ciclev10002362m | 1 | 0.0070 | Chitinase / Poly-beta-glucosaminidase |
| Ciclev10014836m | 1 | 0.0102 | PROTOCHLOROPHYLLIDE-DEPENDENT TRANSLOCON COMPONENT 52, CHLOROPLASTIC |
| Ciclev10029742m | 1 | 0.0006 | EXPRESSED PROTEIN |
| Ciclev10007915m | 1 | 0.0026 | Function not specified |
| Ciclev10021619m | 1 | 0.0009 | Predicted RNA-binding protein SEB4 (RRM superfamily) |
| Ciclev10013265m | 1 | 0.0029 | Function not specified |
| Ciclev10008633m | 1 | 0.0007 | CCCH-type Zn-finger protein |
| Ciclev10016759m | 1 | 0.0002 | Function not specified |
| Ciclev10027245m | 1 | 0.0040 | hAT family C-terminal dimerisation region (Dimer_Tnp_hAT), Domain of unknown function (DUF4371) |
| Ciclev10017331m | 1 | 0.0100 | Function not specified |
| Ciclev10033125m | 1 | 0.0020 | ARF GUANINE-NUCLEOTIDE EXCHANGE FACTOR GNL1-RELATED |
| Ciclev10015831m | 1 | 0.0285 | Function not specified |
| Ciclev10024830m | 1 | 0.0294 | Protein tyrosine kinase (Pkinase_Tyr), Di-glucose binding within endoplasmic reticulum (Malectin) |
| Ciclev10025246m | 1 | 0.0424 | SCARECROW-LIKE PROTEIN 15 |
| Ciclev10011660m | 1 | 0.0228 | WRKY DNA-binding domain (WRKY) |
| Ciclev10006432m | 1 | 0.0257 | X-BOX TRANSCRIPTION FACTOR-RELATED |
| Ciclev10014701m | 1 | 0.0466 | EXPRESSED PROTEIN |
| Ciclev10022207m | 1 | 0.0178 | Function not specified |
| Ciclev10023350m | 1 | 0.0040 | SENTRIN/SUMO-SPECIFIC PROTEASE |
| Ciclev10025377m | 1 | 0.0036 | Flavonoid 3'-monooxygenase / Flavonoid 3'-hydroxylase |
| Ciclev10021708m | 1 | 0.0002 | E3 UBIQUITIN-PROTEIN LIGASE ATL41-RELATED |
| Ciclev10020784m | 1 | 0.0150 | MYELOID LEUKEMIA FACTOR |
| Ciclev10010634m | 1 | 0.0094 | MONOOXYGENASE |
| Ciclev10023387m | 1 | 0.0307 | Function not specified |
| Ciclev10017141m | 1 | 0.0321 | Function not specified |
| Ciclev10032428m | 1 | 0.0342 | CT120 PROTEIN |
| Ciclev10024179m | 1 | 0.0169 | NB-ARC domain (NB-ARC), TIR domain (TIR_2), Leucine rich repeat (LRR_8) |
| Ciclev10005754m | 1 | 0.0343 | Function not specified |
| Ciclev10021861m | 1 | 0.0007 | CHLOROPHYLL A-B BINDING PROTEIN 4, CHLOROPLASTIC |
| Ciclev10001793m | 1 | 0.0240 | Cysteine synthase / OAS sulfhydrylase |
| Ciclev10029278m | 1 | 0.0023 | Function not specified |
| Ciclev10004688m | 1 | 0.0135 | CYCLIC NUCLEOTIDE-GATED ION CHANNEL 11-RELATED |
| Ciclev10027376m | 1 | 0.0090 | Leucine Rich Repeat (LRR) domain-containing protein |
| Ciclev10033097m | 1 | 0.0016 | Function not specified |
| Ciclev10020962m | 1 | 0.0114 | EID1-LIKE F-BOX PROTEIN 3 |
| Ciclev10011986m | 1 | 0.0053 | Function not specified |
| Ciclev10019545m | 1 | 0.0142 | Function not specified |
| Ciclev10011124m | 1 | 0.0012 | STARCH SYNTHASE 2, CHLOROPLASTIC/AMYLOPLASTIC |
| Ciclev10027981m | 1 | 0.0003 | MEDIATOR OF RNA POLYMERASE II TRANSCRIPTION SUBUNIT 37E-RELATED |
| Ciclev10012232m | 1 | 0.0328 | NUCLEAR TRANSCRIPTION FACTOR Y SUBUNIT A-10-RELATED |
| Ciclev10006993m | 1 | 0.0014 | Acireductone dioxygenase (Fe(2+)-requiring) / E-2' |
| Ciclev10007766m | 1 | 0.0002 | Function not specified |
| Ciclev10021323m | 1 | 0.0019 | Function not specified |
| Ciclev10014754m | 1 | 0.0005 | Function not specified |
| Ciclev10001901m | 1 | 0.0350 | GENOMIC DNA, CHROMOSOME 3, P1 CLONE: MQC3 |
| Ciclev10020793m | 1 | 0.0141 | INNER MEMBRANE PROTEIN DEDA-RELATED |
| Ciclev10022170m | 1 | 0.0212 | AXIAL REGULATOR YABBY 1-RELATED |
| Ciclev10005439m | 1 | 0.0265 | Function not specified |
| Ciclev10002711m | 1 | 0.0023 | Function not specified |
| Ciclev10011940m | 1 | 0.0064 | ZINC TRANSPORTER 3-RELATED (1 of 2) |
| Ciclev10029277m | 1 | 0.0028 | POTASSIUM TRANSPORTER 10-RELATED |
| Ciclev10013071m | 1 | 0.0176 | POTASSIUM TRANSPORTER 10-RELATED |
| Ciclev10015446m | 1 | 0.0260 | TRANSCRIPTION ACTIVATOR GLK1-RELATED |
| Ciclev10014344m | 1 | 0.0023 | POTASSIUM TRANSPORTER 10-RELATED |
| Ciclev10000406m | 1 | 0.0008 | POTASSIUM TRANSPORTER 10-RELATED |
| Ciclev10033978m | 1 | 0.0197 | ADP RIBOSYLATION FACTOR-RELATED |
| Ciclev10005644m | 1 | 0.0326 | PROTEIN T04A8.7, ISOFORM A |
| Ciclev10006699m | 1 | 0.0383 | LysM domain (LysM) (1 of 25) |
| Ciclev10016918m | 1 | 0.0189 | PROTEIN PLANT CADMIUM RESISTANCE 11-RELATED (1 of 5) |
| Ciclev10002644m | 1 | 0.0145 | ABA/WDS induced protein (ABA_WDS) (1 of 3) |
| Ciclev10019815m | 1 | 0.0319 | NDP-glucose--starch glucosyltransferase / Waxy protein |
| Ciclev10016286m | 1 | 0.0022 | CHLOROPHYLL A-B BINDING PROTEIN 1, CHLOROPLASTIC-RELATED (1 of 3) |
| Ciclev10014848m | 1 | 0.0123 | PHOSPHOLIPASE A1-IGAMMA3, CHLOROPLASTIC (1 of 1) |
| Ciclev10010866m | 1 | 0.0038 | LEUCINE-RICH REPEAT-CONTAINING PROTEIN (1 of 50) |
| Ciclev10006533m | 1 | 0.0341 | SUGAR TRANSPORT PROTEIN 3 (1 of 16) |
| Ciclev10029982m | 1 | 0.0105 | ALPHA/BETA-HYDROLASES SUPERFAMILY PROTEIN-RELATED (1 of 2) |
| Ciclev10002418m | 1 | 0.0093 | PROTEIN DEHYDRATION-INDUCED 19-RELATED |
| Ciclev10014726m | 1 | 0.0237 | Tetrahydroberberine oxidase / THB oxidase (1 of 46) |
| Ciclev10010502m | 1 | 0.0210 | Adenosylmethionine decarboxylase / S-adenosyl-L-methionine decarboxylase (1 of 4) |
| Ciclev10006837m | 1 | 0.0243 | Pyruvate, phosphate dikinase / Pyruvate, phosphate dikinase |
| Ciclev10031318m | 1 | 0.0007 | PROTEIN DA1-RELATED 2 |
| Ciclev10005583m | 1 | 0.0192 | Ion channel (Ion_trans_2) / Ankyrin repeats (Ank_2) |
| Ciclev10033238m | 1 | 0.0068 | HEAVY METAL TRANSPORT/DETOXIFICATION SUPERFAMILY PROTEIN |
| Ciclev10026395m | 1 | 0.0264 | WRKY DNA -binding domain (WRKY) |
| Ciclev10031327m | 1 | 0.0004 | SERINE CARBOXYPEPTIDASE-LIKE 36-RELATED (1 of 3) |
| Ciclev10018146m | 1 | 0.0074 | Tetrahydroberberine oxidase / THB oxidase (1 of 46) |
| Ciclev10009912m | 1 | 0.0137 | No specific annotation provided |
| Ciclev10025136m | 1 | 0.0081 | ALPHA/BETA-HYDROLASES SUPERFAMILY PROTEIN-RELATED |
| Ciclev10016498m | 1 | 0.0237 | NUCLEAR TRANSCRIPTION FACTOR Y SUBUNIT B-6-RELATED (1 of 2) |
| Ciclev10015847m | 1 | 0.0100 | LYSINE-SPECIFIC DEMETHYLASE 8 |
| Ciclev10002122m | 1 | 0.0072 | SERINE/ARGININE-RICH SPLICING FACTOR 4 |
| Ciclev10006350m | 1 | 0.0172 | PROGRAMMED CELL DEATH 4 (1 of 2) |
| Ciclev10024973m | 1 | 0.0111 | 1-deoxy-D-xylulose-5-phosphate synthase / DXP-synthase |
| Ciclev10015934m | 1 | 0.0076 | PLASTID-LIPID-ASSOCIATED PROTEIN 3, CHLOROPLASTIC-RELATED (1 of 2) |
| Ciclev10017188m | 1 | 0.0345 | VQ MOTIF-CONTAINING PROTEIN |
| Ciclev10020908m | 1 | 0.0336 | UDP-GALACTOSE/UDP-GLUCOSE TRANSPORTER 2 |
| Ciclev10003847m | 1 | 0.0019 | GENOMIC DNA, CHROMOSOME 3, P1 CLONE: MXL8 |
| Ciclev10009153m | 1 | 0.0202 | AMINO ACID PERMEASE 7-RELATED |
| Ciclev10028457m | 1 | 0.0003 | Phytoene desaturase (zeta-carotene-forming) / 2-step phytoene desaturase |
| Ciclev10025089m | 1 | 0.0294 | ZEAXANTHIN EPOXIDASE, CHLOROPLASTIC |
| Ciclev10027761m | 1 | 0.0002 | EXTRA-LARGE GUANINE NUCLEOTIDE-BINDING PROTEIN 1 |
| Ciclev10016229m | 1 | 0.0065 | WRKY TRANSCRIPTION FACTOR 65-RELATED |
| Ciclev10021466m | 1 | 0.0471 | 2-methyl-6-phytyl-1,4-hydroquinone methyltransferase / MPBQ/MSBQ methyltransferase |
| Ciclev10006096m | 1 | 0.0442 | Pathogenesis-related protein Bet v I family (Bet_v_1) |
| Ciclev10023092m | 1 | 0.0287 | No specific annotation provided |
| Ciclev10018024m | 1 | 0.0496 | Protein kinase domain / S-locus glycoprotein domain / D-mannose binding lectin / PAN-like domain |
| Ciclev10009911m | 1 | 0.0026 | No specific annotation provided |
| Ciclev10006871m | 1 | 0.0470 | SUGAR TRANSPORT PROTEIN 3 |
| Ciclev10032044m | 1 | 0.0156 | Stearoyl-[acyl-carrier-protein] 9-desaturase / Stearyl-ACP desaturase |
| Ciclev10027959m | 1 | 0.0082 | DENTIN SIALOPHOSPHOPROTEIN-LIKE PROTEIN |
| Ciclev10020860m | 1 | 0.0291 | Mitochondrial solute carrier protein |
| Ciclev10011624m | 1 | 0.0317 | Glucan 1,4-alpha-glucosidase / Lysosomal alpha-glucosidase |
| Ciclev10029112m | 1 | 0.0133 | Domain of unknown function (DUF3411) (DUF3411) |
| Ciclev10016287m | 1 | 0.0054 | CHLOROPHYLL A-B BINDING PROTEIN 1, CHLOROPLASTIC-RELATED (1 of 3) |
| Ciclev10016074m | 1 | 0.0021 | CHLOROPHYLL A-B BINDING PROTEIN 1, CHLOROPLASTIC-RELATED (1 of 3) |
| Ciclev10032906m | 1 | 0.0199 | ENDOSULFINE |
| Ciclev10033992m | 1 | 0.0122 | No specific annotation provided |
| Ciclev10027000m | 1 | 0.0134 | No specific annotation provided |
| Ciclev10002674m | 1 | 0.0114 | LEUCINE-RICH REPEAT-CONTAINING PROTEIN |
| Ciclev10002010m | 1 | 0.0203 | No specific annotation provided |
| Ciclev10022151m | 1 | 0.0148 | 4EHP, ISOFORM B |
| Ciclev10011171m | 1 | 0.0026 | Protein kinase domain / Leucine Rich Repeat / Leucine rich repeat |
| Ciclev10016523m | 1 | 0.0481 | No specific annotation provided |
| Ciclev10019951m | 1 | 0.0158 | ALDEHYDE DEHYDROGENASE FAMILY 2 MEMBER B7, MITOCHONDRIAL |
| Ciclev10029987m | 1 | 0.0460 | No specific annotation provided |
| Ciclev10031643m | 1 | 0.0002 | MATE EFFLUX FAMILY PROTEIN |
| Ciclev10019811m | 1 | 0.0101 | Cytochrome P450 |
| Ciclev10024952m | 1 | 0.0032 | U-BOX DOMAIN-CONTAINING PROTEIN 50-RELATED |
| Ciclev10018849m | 1 | 0.0010 | HISTONE-LYSINE N-METHYLTRANSFERASE E(Z) |
| Ciclev10018335m | 1 | 0.0037 | NAD(P)-BINDING ROSSMANN-FOLD SUPERFAMILY PROTEIN |
| Ciclev10009329m | 1 | 0.0154 | Nicotinamide-nucleotide adenylyltransferase / NMNAT |
| Ciclev10020105m | 1 | 0.0003 | SENTRIN/SUMO-SPECIFIC PROTEASE |
| Ciclev10022550m | 1 | 0.0092 | RHODANESE-LIKE DOMAIN-CONTAINING PROTEIN 14, CHLOROPLASTIC |
| Ciclev10033591m | 1 | 0.0401 | BIFUNCTIONAL DIHYDROCAMALEXATE SYNTHASE/CAMALEXIN SYNTHASE |
| Ciclev10009966m | 1 | 0.0250 | DYNEIN LIGHT CHAIN TYPE 1 FAMILY PROTEIN |
| Ciclev10007341m | 1 | 0.0413 | Leucine Rich Repeat (LRR_1) / Leucine rich repeat N-terminal domain (LRRNT_2) / Leucine rich repeat (LRR_8) |
| Ciclev10002749m | 1 | 0.0171 | ABA/WDS induced protein (ABA_WDS) |
| Ciclev10002269m | 1 | 0.0073 | No specific annotation provided |
| Ciclev10009975m | 1 | 0.0169 | Eukaryotic translation initiation factor SUI1 |
| Ciclev10028830m | 1 | 0.0006 | No specific annotation provided |
| Ciclev10002957m | 1 | 0.0068 | No specific annotation provided |
| Ciclev10026553m | 1 | 0.0330 | Kelch repeat domain |
| Ciclev10026592m | 1 | 0.0199 | ADP ribosylation factor-related |
| Ciclev10001814m | 1 | 0.0006 | No specific annotation provided |
| Ciclev10031167m | 1 | 0.0079 | CBS domain-containing protein CBSCBSPB4-related |
| Ciclev10017662m | 1 | 0.0156 | C2 domain |
| Ciclev10008672m | 1 | 0.0017 | No specific annotation provided |
| Ciclev10007003m | 1 | 0.0017 | Acireductone dioxygenase (Fe(2+)-requiring) |
| Ciclev10011612m | 1 | 0.0286 | Inositol 5-phosphatase |
| Ciclev10033006m | 1 | 0.0306 | No specific annotation provided |
| Ciclev10010467m | 1 | 0.0468 | PB1 domain (PB1) |
| Ciclev10026533m | 1 | 0.0341 | No specific annotation provided |
| Ciclev10009102m | 1 | 0.0258 | No specific annotation provided |
